# Estimating the time course of biomarker changes in Alzheimer’s disease

**DOI:** 10.1093/brain/awaf413

**Published:** 2025-11-03

**Authors:** Lars Lau Raket, Alexa Pichet Binette, Niklas Mattsson-Carlgren, Shorena Janelidze, Henrik Zetterberg, Nicholas J Ashton, Kaj Blennow, Erik Stomrud, Sebastian Palmqvist, Oskar Hansson

**Affiliations:** Clinical Memory Research Unit, Department of Clinical Sciences in Malmö, Lund University, Lund 223 62, Sweden; Eli Lilly and Company, Indianapolis, IN 46285, USA; Clinical Memory Research Unit, Department of Clinical Sciences in Malmö, Lund University, Lund 223 62, Sweden; Clinical Memory Research Unit, Department of Clinical Sciences in Malmö, Lund University, Lund 223 62, Sweden; Department of Neurology, Skåne University Hospital, Lund University, Lund 221 85, Sweden; Wallenberg Center for Molecular Medicine, Lund University, Lund 223 62, Sweden; Clinical Memory Research Unit, Department of Clinical Sciences in Malmö, Lund University, Lund 223 62, Sweden; Department of Psychiatry and Neurochemistry, Institute of Neuroscience and Physiology, the Sahlgrenska Academy at the University of Gothenburg, Mölndal 431 39, Sweden; Clinical Neurochemistry Laboratory, Sahlgrenska University Hospital, Mölndal 431 39, Sweden; Department of Neurodegenerative Disease, UCL Institute of Neurology, London WC1N 3BG, UK; UK Dementia Research Institute at UCL, London NW1 3BT, UK; Hong Kong Center for Neurodegenerative Diseases, Clear Water Bay, Hong Kong 999077, China; Wisconsin Alzheimer’s Disease Research Center, University of Wisconsin School of Medicine and Public Health, University of Wisconsin-Madison, Madison, WI 53792, USA; Department of Psychiatry and Neurochemistry, Institute of Neuroscience and Physiology, the Sahlgrenska Academy at the University of Gothenburg, Mölndal 431 39, Sweden; Banner Alzheimer’s Institute and University of Arizona, Phoenix, AZ 85006, USA; Banner Sun Health Research Institute, Sun City, AZ 85351, USA; Department of Psychiatry and Neurochemistry, Institute of Neuroscience and Physiology, the Sahlgrenska Academy at the University of Gothenburg, Mölndal 431 39, Sweden; Clinical Neurochemistry Laboratory, Sahlgrenska University Hospital, Mölndal 431 39, Sweden; Clinical Memory Research Unit, Department of Clinical Sciences in Malmö, Lund University, Lund 223 62, Sweden; Memory Clinic, Skåne University Hospital, Malmö 211 46, Sweden; Clinical Memory Research Unit, Department of Clinical Sciences in Malmö, Lund University, Lund 223 62, Sweden; Memory Clinic, Skåne University Hospital, Malmö 211 46, Sweden; Clinical Memory Research Unit, Department of Clinical Sciences in Malmö, Lund University, Lund 223 62, Sweden; Memory Clinic, Skåne University Hospital, Malmö 211 46, Sweden

**Keywords:** Alzheimer’s disease, biomarkers, disease progression, CSF, PET, amyloid-beta

## Abstract

Recent advancements in biomarkers have transformed Alzheimer’s disease (AD) diagnosis from being purely symptom-based to include biological criteria. With new treatments targeting the core biology of Alzheimer’s disease, understanding the timeline of biological changes is crucial as the disease progresses over decades.

Longitudinal data from amyloid-beta (Aβ) PET and cognitive tests [Mini-Mental State Examination (MMSE) and Alzheimer's Disease Assessment Scale–Cognitive Subscale (ADAS-cog)] from the Alzheimer’s Disease Neuroimaging Initiative (*n* = 1448) and BioFINDER (*n* = 2088) were used to stage patients against an estimated continuous disease timeline (predicted time since Aβ-PET positivity). The estimated timeline was validated by comparing correlations with unseen biomarkers and cognitive measures against alternative staging approaches. Trajectories for plasma, CSF, MRI and PET biomarkers, measuring Aβ, tau and neurodegeneration, were mapped along this Alzheimer’s disease continuum.

The proposed staging approach was found to produce stronger correlations with unseen cognitive measures and biomarkers compared to alternative staging methods, including amyloid and tau PET clocks (all pairwise *P* < 0.05). Findings related to biomarker trajectories were highly consistent across cohorts. The period from Aβ-PET positivity to end-stage Alzheimer’s disease dementia (MMSE = 0) was estimated at 20–25 years, with a presymptomatic phase of 7–11 years. CSF Aβ_42_/Aβ_40_ became abnormal about a year before Aβ-PET positivity, CSF phosphorylated-tau (p-tau)231, p-tau217 and plasma phosphorylated/neuritic plaque-tau217 1–3 years after, and tau-PET about 8 years after. Neurodegenerative biomarkers, such as hippocampal volume, became clearly abnormal in early dementia stages, 14–16 years after Aβ-PET positivity.

The progression from initial biomarker abnormality to severe Alzheimer’s disease spans two decades. Disease progression modelling elucidates the evolution of AD biomarkers and cognition, highlighting the relative timing of biomarker abnormalities. These models can determine disease stages, aiding in prognosis and the evaluation of disease-modifying treatments.

## Teaser

Raket *et al.* study the time course of biomarker changes in Alzheimer's disease, from the earliest detectable changes—often appearing a decade prior to symptom onset—to end-stage dementia. Understanding when biomarkers typically become abnormal can help in determining disease stages and evaluating disease-modifying treatments.

## Introduction

Alzheimer’s disease (AD) is biologically defined by the abnormal presence of amyloid-beta (Aβ) plaques and tau-containing neurofibrillary tangles in the brain. AD progresses slowly, starting with a preclinical (presymptomatic) phase where the pathological hallmarks are present with no objectively verified cognition symptoms. This preclinical phase has been suggested to last up to several decades.^1^ The preclinical phase is followed by a prodromal phase where cognitive symptoms emerge and increase in severity, followed by dementia, where patients lose their ability to independently perform activities of daily living.

Over the past few decades, a wide range of biomarkers for AD have become available. These include assays to measure Aβ and tau proteins in CSF, PET imaging of AD proteinopathies, volumetric MRI and accurate blood-based biomarkers reproducing CSF findings.^2^ Currently available biomarkers enable precise differential diagnosis of AD and provide staging and prognostic information.^2-5^ Based on animal studies, neuropathology and longitudinal biomarker studies, a hypothetical biomarker cascade following the Alzheimer’s pathological cascade has been proposed.^6^ This model suggests that the prototypical disease trajectory is characterized by initial Aβ plaque accumulation in the brain, followed by spreading of tau pathology and neurodegeneration, which in combination leads to cognitive symptoms. This Alzheimer’s biomarker cascade has been largely validated and refined in many previous studies.^7,8^ However, some key unknowns remain. The temporal aspects of AD in relation to biomarker changes have been underappreciated, with most studies either relating biomarkers to other biomarkers^8^ or relating them to coarse disease stages based on symptom severity evaluated at single time points.^9^ Typical groupings [cognitively unimpaired (CU), mild cognitive impairment (MCI), dementia] represent stages that can last many years, with a large severity span within groups, with ambiguous differentiation between groups, and which can be influenced by co-pathologies and premorbid cognitive capacity. Such staging does not reflect the continuous progressive nature of AD, and therefore there are still many unknowns around the fine-scale time evolution of AD.

The current understanding of the evolution of AD in continuous time has relied heavily on studies in autosomal dominant AD. A key reason for this is that knowledge of a subject’s mutation type and other characteristics can be used to roughly predict age of symptom onset and progression pattern.^10^ This enables calculation of a subject-level disease timescale, which in turn enables continuous-time population-level modelling of biomarker trajectories.^11,12^ This approach has been instrumental in understanding not only autosomal dominant disease, but also sporadic AD, which has many pathophysiological similarities, but also some differences that hamper this extrapolation.^13^

Several methods have been proposed to enable better modelling of biomarker trajectories in sporadic AD. Biologically consistent estimates of biomarker trajectories along Aβ or tau accumulation, as measured by PET, have been reported.^8,14,15^ To derive a continuous timeline, mimicking the time to symptom onset construct in autosomal dominant AD, amyloid and tau clocks that map PET signals to time, and a range of different latent-time disease progression models using both biomarkers and clinical data have been proposed.^16-22^ As opposed to the estimated time to symptom onset in autosomal dominant AD, these approaches make use of patterns observed in longitudinal clinical and/or biomarker data to dynamically derive a new latent timescale on which the longitudinal observations are aligned.^23^

In this paper, we propose a disease progression model that utilizes both clinical and Aβ PET data to model the time course of AD over a latent timescale that represents predicted time since Aβ-PET positivity. We apply the model in well-characterized participants who are either Aβ-negative CU or have biomarker-confirmed AD (all disease stages) in two large cohorts: the Alzheimer’s Disease Neuroimaging Initiative (ADNI) and the Swedish BioFINDER study. Based on the modelling, continuous-time disease stages representing time since Aβ PET positivity were predicted for all subjects, which allowed continuous and time-consistent modelling of biomarker trajectories along the Alzheimer’s continuum. We report trajectories for a wide selection of biomarkers measuring aspects of amyloidosis, tauopathy, neurodegeneration and inflammation along predicted time since Aβ positivity, including CSF Aβ_42_/Aβ_40_ and Aβ PET, tau PET, various phosphorylated-tau (p-tau) species in CSF and plasma, CSF/plasma neurofilament light (NfL) and volumetric MRI, plasma glial fibrillary acidic protein (GFAP) and CSF sTREM2. These findings shed new light on the fine-scale time evolution of AD.

## Materials and methods

### Participants

This study included participants from ADNI, recruited under protocols 1, GO, 2 and 3 (ClinicalTrials.gov ID: NCT00106899, NCT01078636, NCT01231971 and NCT02854033) and participants from the Swedish BioFINDER study, recruited under protocols 1 and 2 (NCT01208675 and NCT03174938). All participants in both studies provided written informed consent, and the studies were approved by the appropriate ethical review authorities.

ADNI is a multi-site study launched in 2003 as a public-private partnership. The primary goal of ADNI has been to test whether serial MRI, PET, other biological markers, and clinical and neuropsychological assessment can be combined to measure the progression of MCI and early AD. For up-to-date information, see www.adni-info.org.

BioFINDER-1 followed CU, MCI and dementia participants recruited between 2009 and 2014 for up to 10 years. BioFINDER-2 is an ongoing longitudinal study including participants across the full spectrum of AD, which started in 2017.

The current study used ADNI data collected from 2005 to 24 August 2023, all BioFINDER-1 data from 2009 to 12 December 2023 and BioFINDER-2 data collected from 2017 to 24 January 2024.

#### Inclusion criteria

The inclusion and exclusion criteria for ADNI are described in the study protocols available on the ADNI webpage, and inclusion and exclusion criteria for the BioFINDER studies have been previously described (NCT01208675 and NCT03174938).^24-26^ Briefly summarized, in the BioFINDER studies, CU participants did not fulfil the National Institute on Aging and Alzheimer's Association criteria for MCI or dementia^27^ (performed within normal ranges on a large cognitive test battery) and were between 40 and 100 years old. Cognitively impaired participants had been referred to the participating memory clinics at Skåne University Hospital or Ängelholm Hospital in Sweden. Participants with MCI did not fulfil the Diagnostic and Statistical Manual of Mental Disorders, fifth edition (DSM-5) criteria for major cognitive disorder^28^ and performed below normal ranges in at least one cognitive domain as previously described for BioFINDER-1^29^ and for BioFINDER-2.^26^ All participants understood Swedish to the extent that an interpreter was not necessary.

For the present study, we included participants who at baseline were at least 50 years of age and had a valid assessment of their cognitive status [ADNI: unimpaired, significant memory concern, early MCI, late MCI, dementia; BioFINDER: unimpaired, subjective cognitive decline (SCD), MCI, dementia], a valid assessment of Aβ status (see details later) and at least one measurement of Aβ PET, Mini-Mental State Examination (MMSE) or Alzheimer's Disease Assessment Scale–Cognitive Subscale (ADAS-cog).

In BioFINDER, symptomatic participants with an established primary aetiology other than AD were excluded. Aetiology was assessed based on a thorough longitudinal clinical evaluation, biomarker information and in some cases, genetic information. These clinical evaluation criteria have been described previously.^24^ Patients were only excluded based on the clinically established primary aetiology, meaning that no effort was made to exclude AD patients with co-pathologies.

CU Aβ-negative (Aβ−) and Aβ-positive (Aβ+) participants were included. Since the goal was to estimate AD-specific trajectories, participants were excluded if they had negative Aβ biomarkers at visits where they showed objective cognitive impairment [diagnosis of MCI or dementia or Cognitive Dementia Rating (CDR) global score >0 or MMSE < 26].

#### Aβ status

Aβ status (Aβ+/Aβ−) was determined using CSF or PET at every visit where either was available. For CSF, Aβ-positivity was assessed by the Aβ_42_/Aβ_40_ ratio. In ADNI, Aβ_42_ and Aβ_40_ levels were measured on a cobas e 601 system using Roche Elecsys immunoassays (Aβ_42_/Aβ_40_ positivity cut-off <0.0666) or by 2D-ultra high-performance liquid chromatography-tandem mass spectrometry^30^ (Aβ_42_/Aβ_40_ positivity cut-off <0.138). In BioFINDER, Aβ_42_ and *c were analysed on a cobas e 601 system using the Roche NeuroToolKit, and the cut-off for Aβ-positivity was <0.066 in BioFINDER-1^31^ and <0.080 in BioFINDER-2.^32^ The PET tracers ^18^F-florbetapir, ^18^F-florbetaben and ^11^C-Pittsburgh Compound B were used to establish Aβ-PET positivity in ADNI using published cut-offs by the ADNI PET core, defined by standardized uptake value ratio (SUVR) computed in a composite cortical region referenced to the cerebellum. Cut-offs were 1.11 SUVR for ^18^F-florbetapir and 1.08 SUVR for ^18^F-florbetaben and 1.22 for ^11^C-Pittsburgh Compound B.^33,34^ In BioFINDER, Aβ-PET positivity was established using ^18^F-flutemetamol SUVR in a composite cortical region referenced to the cerebellum with a cut-off of 1.03.^35^

Negative Aβ status was carried backwards, so individuals without a valid Aβ status at baseline but a post-baseline Aβ− status were assumed Aβ− at baseline. Aβ+ status was carried forward.

In some cases, both CSF and PET Aβ biomarkers were available and produced discrepant results (ADNI 229/1954 visits; BioFINDER 271/1078 visits). Discrepant results were primarily in the form of Aβ+ status on CSF and Aβ− status on PET (ADNI 142/229; BioFINDER 217/271) and mostly observed in CU subjects (ADNI 126/229; BioFINDER 196/271), consistent with previous observations that CSF Aβ biomarkers typically become abnormal before PET Aβ biomarkers.^8,36^ For CU subjects, we assumed Aβ-positivity if just one of the biomarkers was abnormal. For cognitively impaired subjects, we required Aβ-PET positivity.

### Cognitive, functional and clinical outcomes

We used longitudinal MMSE and ADAS-cog^37^ total scores for the disease progression modelling in both cohorts. In ADNI, the 13-item version of ADAS-cog was used, while in BioFINDER, a two-item version (including immediate and delayed recall) was used.

For exploring the construct validity of the estimated timeline, we used longitudinal scores from the CDR-Sum of Boxes (CDR-SB) and Trail Making B in both cohorts. In ADNI, we further used logical memory delayed recall scores.

For validation analyses and for describing the clinical stages associated with the disease progression model analyses, we used clinical diagnosis (CU, MCI, dementia) at each visit. In BioFINDER, clinical diagnosis was not consistently available at all follow-up visits, so we imputed missing diagnoses at visits where we had high certainty of the diagnosis using the following imputation rules: when visits before and after the missing visit had the same diagnosis, all in-between visits with missing diagnosis were interpolated to have the same diagnosis. Cognitively unimpaired status was carried backwards, and diagnoses of dementia were carried forward. When available, the CDR global score (0 = CU, 0.5 = MCI, ≥1 = dementia) was used to impute clinical diagnosis at the remaining visits with missing diagnosis.

### Plasma, CSF and imaging biomarkers

The following section describes the biomarkers used for disease progression modelling, validation or estimation of biomarker trajectories across the AD continuum. The availability of individual biomarkers differed between studies and subjects. Sample sizes of available biomarker data will be stated in the analyses.

#### Plasma

In ADNI, included plasma biomarkers were p-tau181, NfL and GFAP, all measured by an electrochemiluminescence immunoassay on the fully automated cobas e 601 (Roche Diagnostics NeuroToolKit),^38^ p-tau217, p/np-tau217, defined as the ratio of p-tau217 to neuritic plaque-tau217 (np-tau217) and Aβ_42_/Aβ_40_ measured by liquid chromatography–tandem high-resolution mass spectrometry analysis (PrecivityAD2).^39^

In BioFINDER, the included plasma biomarkers were NfL measured using the Roche Diagnostics NeuroToolKit described above, GFAP measured using a single molecule array (Simoa)-based assay,^40^ p-tau181, p/np-tau181, p-tau217, p/np-tau217, Aβ_42_/Aβ_40_ measured by liquid chromatography–tandem high-resolution mass spectrometry analysis^38,41^ and p-tau231 measured using a Simoa developed at the University of Gothenburg.^42^

#### CSF

In ADNI, the included CSF biomarkers were Aβ_42_ and Aβ_42_/Aβ_40_, p-tau181 and p-tau181/Aβ_42_ ratio measured on a cobas e 601 system using Roche Elecsys immunoassays,^43^ NfL measured with an ELISA from UmanDiagnostic AB,^44^ sTREM2 and neurogranin measured with immunoassays using the Meso Scale Discovery (MSD) platform^45,46^ and YKL-40 measured using the MicroVue YKL-40 ELISA.^47^

In BioFINDER, the included CSF biomarkers were Aβ_42_, Aβ_42_/Aβ_40_, p-tau181, p-tau181/Aβ_42_ ratio, total tau, NfL, neurogranin, sTREM2 and YKL-40, all measured on a cobas e 601 system using the Roche NeuroToolKit. Furthermore, BioFINDER included CSF p-tau217 measured on the MSD platform using an assay developed by Eli Lilly,^48^ and CSF p-tau231 measured on the Simoa platform developed at University of Gothenburg using an antibody from ADx NeuroSciences.^49^

#### Imaging

In ADNI, the included imaging biomarkers were Aβ-PET Centiloid computed for the tracers ^11^C-Pittsburg Compound B, ^18^F-flortaucipir and ^18^F-florbetaben, based on the SUVR ratio in a composite cortical region of interest normalized to the whole cerebellum,^50^ tau-PET SUVR using ^18^F-flortaucipir in Braak regions I, III-IV and V-VI normalized to inferior cerebellar grey matter uptake,^51^  ^18^F-FDG PET SUVR in a meta region of interest,^52^ MRI biomarkers of hippocampal and ventricular volume normalized to whole-brain volume^53^ and a composite cortical thickness AD-signature,^54^ all derived using FreeSurfer.

In BioFINDER, the included biomarkers were Aβ-PET SUVR using ^18^F-flutemetamol in a composite cortical region normalized to the cerebellum,^55^ tau-PET SUVR using ^18^F-RO948 in Braak regions I, III-IV and V-VI normalized to the inferior cerebellum grey matter,^55^ MRI biomarkers of hippocampal and ventricular volume normalized to whole-brain volume and a composite cortical thickness AD-signature, all derived using FreeSurfer.

### Statistical analysis

#### Disease progression modeling

We developed a semiparametric extension of the multivariate latent-time disease progression model described by Kühnel *et al*.^56^ which estimates trajectories of outcome measures by aligning multivariate subject-level outcome trajectories. Unlike many other latent-time disease progression models, this modelling approach estimates mean trajectories in a time-consistent manner by using relative visit timings for individual subjects as a scaffold for time-based estimation. Consequently, a change in values of the mean trajectory associated with a change in time unit (e.g. 1 year) is reflective of the typical change observed over the same time unit for subjects that were matched to that part of the trajectory. The first step in estimating a time-consistent biomarker cascade was to estimate a continuous-time disease trajectory for selected outcomes simultaneously. This involved predicting the continuous-time progression of each subject along this estimated multivariate disease trajectory. The estimated multivariate disease trajectory was based on longitudinal measures of Aβ PET, MMSE and ADAS-cog. The inclusion of both an Aβ biomarker and clinical scales ensures adequate staging information in both pre-symptomatic and symptomatic stages of AD.

The model was defined as follows. Let $y_{ijk}$ denote subject *i*’s observation of the *k*th outcome measure $t_{ij}$ years after the baseline visit. The mean trajectory $\theta_{k}$ of the *k*th outcome over the disease continuum was estimated from the model


(1) $$y_{ijk}=\theta_{k}(t_{ij}+S_{\mathrm{bl}\;\;\mathrm{status}(i)}+S_{\mathrm{bl}\;A-(i)}+S_{i})+x_{ik}+e_{ijk}$$

where we will refer to the time argument $t_{ij}+S_{\mathrm{bl}\;\mathrm{status}(i)}+S_{\mathrm{bl}\;A-(i)}+S_{i}$ that is shared across outcomes as ‘disease time’.

The model parameters were modelled as follows:

-
$\theta_{k}$ was a monotone Hermite spline with five degrees of freedom. The spline had 5 + 2 knots. Relative to average baseline disease time of the cognitively normal Aβ+ group, the five internal knots were placed at −8.33, 0, 8.33, 16.67 and 25 years with two additional knots replicating the boundary values placed 1 month before and after these respective knots to limit boundary artefacts.
-
$S_{\mathrm{bl}\;\mathrm{status}(i)}$ was a fixed effect time-shift describing the average shift in disease time of the baseline status group of subject *i* (i.e. CU, SCD, MCI or dementia in the BioFINDER study).
-
$S_{\mathrm{bl}\;A-(i)}$ was a fixed effect time-shift describing the average shift in disease time associated with Aβ− status at baseline.
-
$S_{i}$ was a random effect time-shift describing the time deviation of subject *i* relative to their baseline group and Aβ+/Aβ− status.
-
$x_{ik}$ was a random effect intercept describing subject *i*’s consistent deviation in outcome measure *k*, for example the consistent deviation observed for patients with comorbidities or low education that score worse on clinical scales compared to their AD progression stage, an unstructured covariance matrix was used to model the correlation across outcomes.
-
$e_{ijk}$ was independent identically distributed Gaussian noise with separate variance parameters for each outcome *k*.

**Figure 1:**
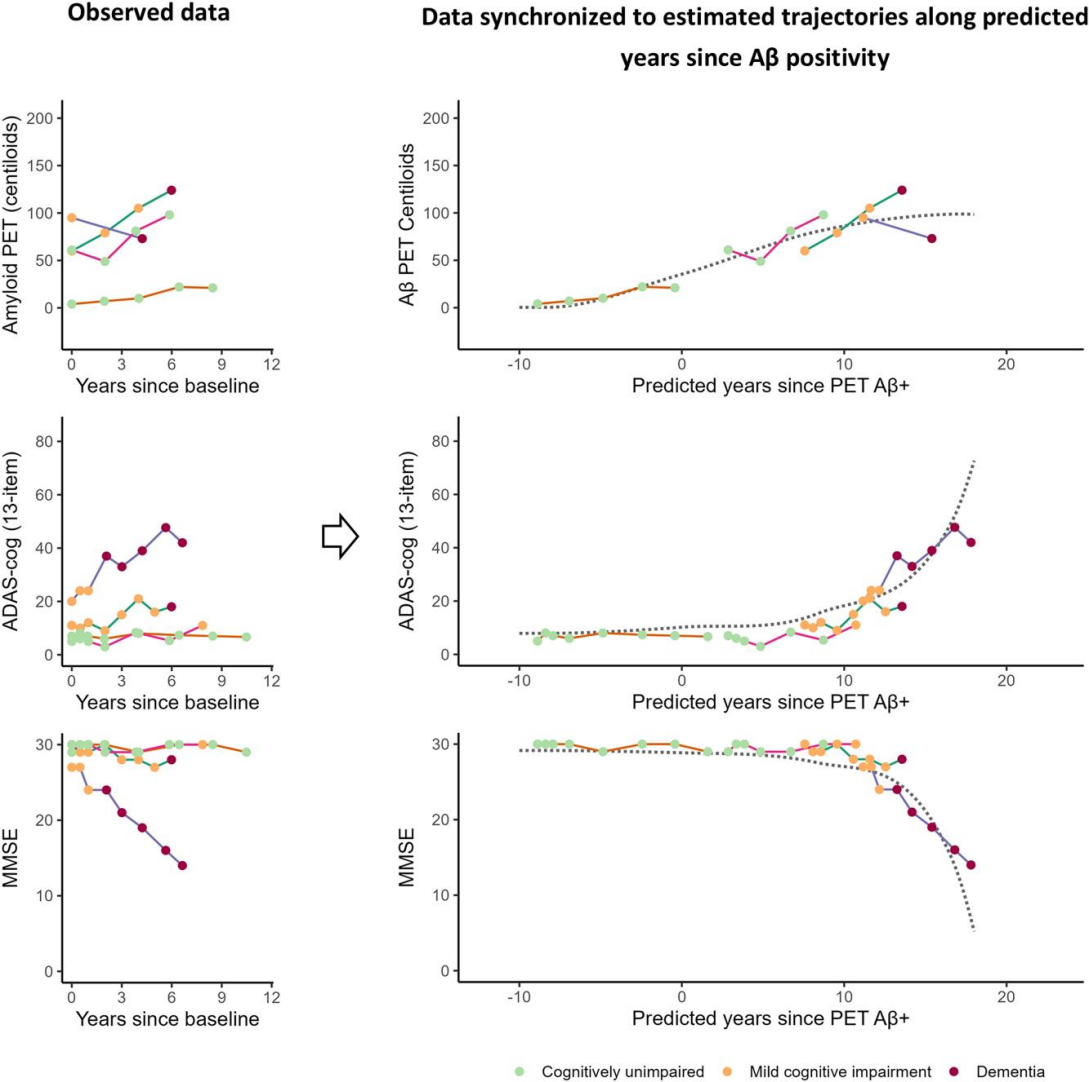
**Estimated trajectories of outcome**. Illustration shows the alignment of observed samples from four subjects against the estimated trajectories of outcomes (dotted lines) along the predicted disease timescale. The line colours differentiate individual subjects, and the colours of the filled circles indicate the diagnosis of a subject at a given visit. The dotted mean trajectories are estimated simultaneously with the alignment of individual subject trajectories based on all available data. Aβ = amyloid-β; ADAS-cog = Alzheimer's Disease Assessment Scale-Cognitive Subscale; MMSE = Mini-Mental State Examination.

To avoid overparameterization, the fixed effect time shifts were anchored such that a disease time of 0 was the time at which a subject on average reached Aβ+ status measured by PET. The predicted disease time thus represents predicted years since Aβ PET positivity. The estimation process is illustrated in Fig. 1. All parameters in the model were estimated using maximum likelihood estimation. The maximum a posteriori criterion was used for prediction of random effects. Code for fitting the model is available in the *progmod* R package.^57^

### Model validation

The construct validity of the disease progression model was assessed internally in ADNI and BioFINDER based on the predicted disease time’s correlation to longitudinal scores from clinical scales measuring cognition and function (Trail Making B, Logical memory delayed recall, CDR-SB) and biomarkers related to Aβ, tau and neurodegeneration that were not included in the model. The individual scales and biomarkers were previously described, and biomarkers were grouped into Aβ, tau and neurodegeneration. The absolute pairwise Spearman correlations to these scales and biomarkers, as well as the domain-weighted average (average of average absolute pairwise Spearman correlation within each of the four domains: cognition and function, Aβ, tau, neurodegeneration), were compared to alternative longitudinal staging variables related to disease progression. These staging variables included age, clinical diagnosis (0 = CU, 1 = MCI, 2 = dementia), MMSE, ADAS-cog and Aβ PET. Furthermore, three separate analyses in ADNI compared predicted time since Aβ PET positivity to recently published amyloid and tau clocks estimated based on longitudinal Aβ PET and tau PET data following the algorithm outlined by Milà-Alomà and colleagues,^21^ and to the two established continuous-time disease progression models GRACE^17^ and LTJMM^58^ with models fitted on the same variables as the proposed model based on published implementations. Pairwise comparisons between predicted time since Aβ PET positivity and all other staging measures were done by comparing absolute correlation across all scales and biomarkers with a binomial sign test. The analysis only included visits where all staging variables were available to ensure comparability (staging variable assigned to the same visit but not required to be collected on the same day, e.g. there were often several weeks’ difference between clinical scale data collection and PET scans).

### Estimating biomarker trajectories

Based on Model (1), each subject had their time since Aβ-PET positivity predicted at all time points. Biomarker trajectories were then analysed along this predicted disease timeline using a robust quantile mixed-effects spline model with a random Laplace distributed intercept within participants to estimate the median biomarker trajectory.^59^ Natural cubic splines with degrees of freedom ranging from 0 (no time dependence), 1 (linear slope) and up to 8 were used to parametrize each median biomarker trajectory, and the model with the lowest Bayesian Information Criterion (BIC) was selected. These biomarker trajectories were normalized against the median and 95% percentile (in the direction of abnormality) of the biomarker values of CU Aβ− individuals. The empirical case bootstrap (1000 resamplings) was used to calculate 95% confidence intervals of when individual biomarkers crossed 95% abnormality percentiles.

## Results

### Study participants

In ADNI, 1963 subjects fulfilled the inclusion criteria, and 2277 subjects from BioFINDER fulfilled the inclusion criteria. Among these subjects, 515 subjects in ADNI and 189 subjects in BioFINDER were excluded based on the criteria related to having concurrent Aβ− status and objective cognitive impairment. The baseline characteristics of the 1448 ADNI subjects and 2088 BioFINDER subjects included in the present study are given in Table 1.

**Table 1:** Baseline characteristics of subjects in ADNI and BioFINDER

	ADNI	BioFINDER
*N*	1448	2088
Females, *n* (%)	728 (50%)	1199 (57%)
Education, years	16 [14, 18]	12 [10, 15]
Age, years	72.9 [68.1, 77.9]	72.4 [66.6, 76.9]
Follow-up time, years	2.8 [1.0, 4.3]	3.8 [1.6, 6.2]
**Cognitively unimpaired at baseline**		
*N*	677	1436
Female, *n* (%)	395 (58%)	855 (60%)
APOE ɛ4 carriers, *n* (%)	200 (32%)	530 (37%)
Education, years	16 [15, 18]	12 [10, 15]
Age, years	71.4 [67.1, 76.3]	71.4 [65.0, 76.4]
Aβ-positive, *n* (%)	293 (43%)	499 (35%)
MMSE	29 [29, 30]	29 [28, 30]
**MCI at baseline**		
*N*	501	400
Female, *n* (%)	212 (42%)	202 (50%)
APOE ɛ4 carriers, *n* (%)	314 (66%)	286 (72%)
Education, years	16 [14, 18]	12 [9, 15]
Age, years	73.9 [68.8, 78.2]	73.8 [69.3, 77.2]
Aβ-positive, *n* (%)	478 (100%)	400 (100%)
MMSE	28 [26, 29]	27 [25, 28]
**Dementia at baseline**		
*N*	270	252
Female, *n* (%)	121 (45%)	142 (56%)
APOE ɛ4 carriers, *n* (%)	194 (75%)	179 (71%)
Education, years	16 [13, 18]	12 [9, 14]
Age, years	74.4 [69.1, 79.6]	75.1 [70.7, 78.5]
Aβ-positive, *n* (%)	265 (100%)	252 (100%)
MMSE	23 [21, 25]	21 [18, 24]

Continuous measures are given as median [interquartile range]. ADNI = Alzheimer’s Disease Neuroimaging Initiative; Aβ = amyloid-β; MMSE = Mini-Mental State Examination.

### Disease progression modeling results and validation

**Figure 2:**
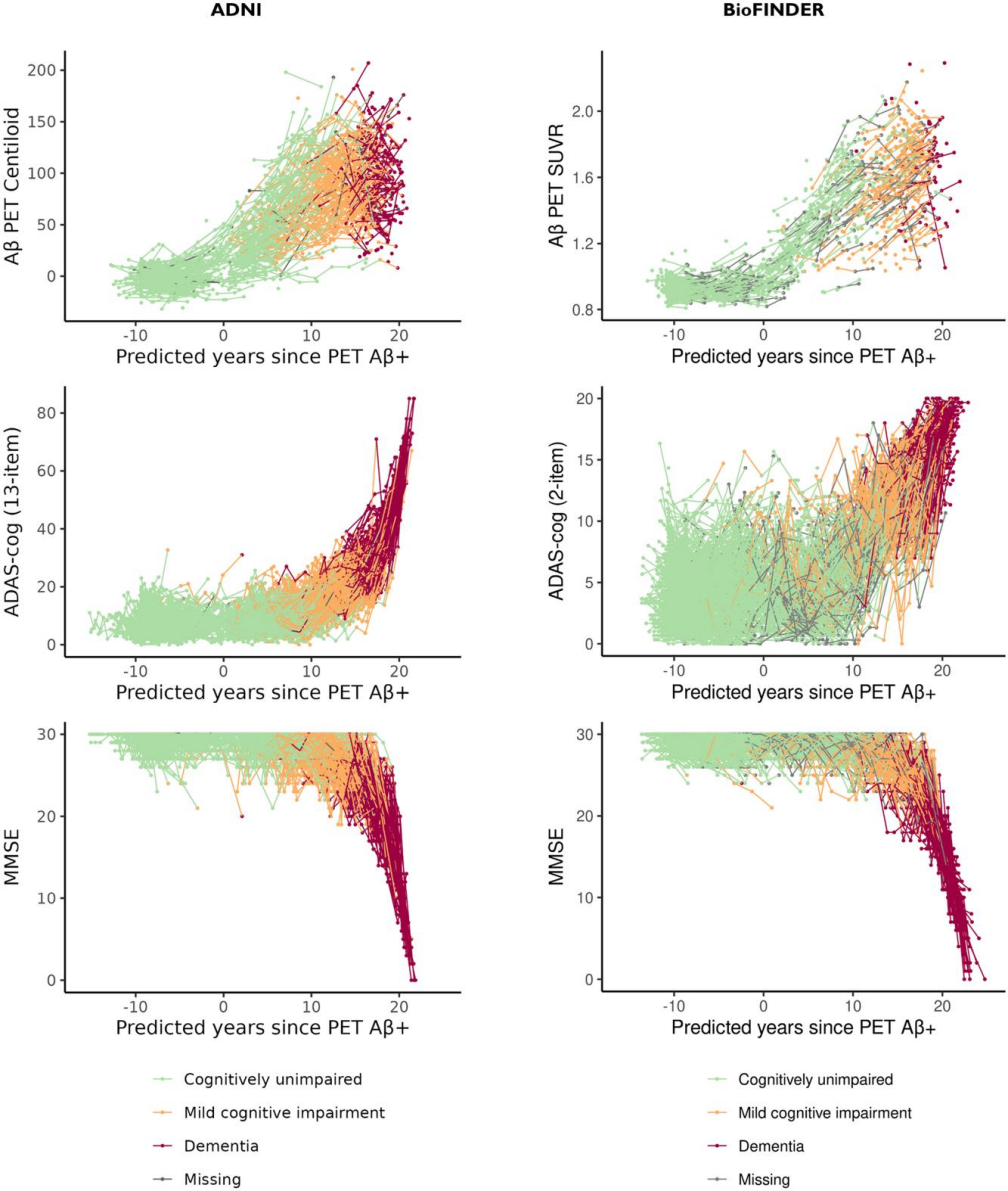
**Participants’ longitudinal trajectories of the three measures used to build the disease progression model.** Aβ PET, ADAS-cog and MMSE in ADNI (*N* = 1448) and BioFINDER (*N* = 2088) plotted against predicted time since Aβ-PET positivity. The timescale is measured in years with 0 is anchored at the time of average Aβ positivity as assessed by PET. Aβ = amyloid-β; ADAS-cog = Alzheimer's Disease Assessment Scale-Cognitive Subscale; ADNI = Alzheimer’s Disease Neuroimaging Initiative; MMSE = Mini-Mental State Examination; SUVR = standardized uptake value ratio.

The observed trajectories of the outcome measures plotted against the predicted years since PET Aβ-positivity in the two cohorts are shown in Fig. 2. There were substantial overlaps between longitudinal clinical diagnoses stratified by Aβ status along the predicted disease timescale, but the distributions were very similar between cohorts (Supplementary Figs 1 and 2). Sensitivity analyses using only available diagnoses in BioFINDER (data not shown) suggested that the imputation strategy for missing diagnoses in BioFINDER did not affect the estimated distribution of diagnoses along the predicted disease timescale.

Inspection of conditional residuals indicated a symmetric distribution along the predicted disease timeline. However, we observed heterogeneous variance in both cohorts, which increased with disease age (Supplementary Figs 3 and 4). This indicates that there is variability in the datasets not fully accounted for by the model. However, we believe this deviation does not significantly impact the conclusions of our subsequent analyses, as it is more likely to increase uncertainty rather than introduce a meaningful bias. Comparison of the proposed model with sub-models excluding random effects suggested that both the random time shifts and random intercepts captured a very substantial amount of variation in the data. Most notably, excluding the random shift parameter $S_{i}$ (one degree of freedom) increased the estimated residual variance in ADNI by 9% for Aβ-PET Centiloid, 113% for ADAS-cog (13-item) and 211% for MMSE (Supplementary Table 1). In BioFINDER, the increases in estimated residual variance associated with excluding $S_{i}$ were 10% for Aβ-PET SUVR, 12% for ADAS-cog (2-item) and 94% for MMSE (Supplementary Table 2).

The correlations between the predicted disease time, alternative staging variables and unseen validation variables in ADNI and BioFINDER are given in Tables 2 and 3, respectively. Comparisons of correlations for predicted disease time and an amyloid clock and a tau clock are given in Supplementary Table 3, and comparisons to other disease progression models are given in Supplementary Table 4. To test the validity of the predicted disease time as a single continuous staging measure that effectively captures overall disease progression, the simultaneous pattern of correlations to the full set of validation variables was compared between predicted disease time and the alternative staging variables. In both cohorts, the predicted disease time showed a significantly stronger pattern of correlation to the set of validation variables compared to all other staging variables (all pairwise *P* < 0.05; binomial sign test), including the amyloid PET clock (*P =* 0.0034) and the tau PET clock in (*P* = 0.0002) in ADNI. The predicted disease showed numerically stronger average correlations than GRACE disease time (*P* = 0.1796) and LTJMM disease time (*P* = 0.0001). Notably, predicted disease time and GRACE disease time showed similar correlations for validation variables in the cognition and function and neurodegeneration categories, while predicted disease time showed markedly stronger correlations across all validation variables in the Aβ and tau categories. We note that while predicted disease time had the strongest correlations as a single measure, the validation variables may be affected by multiple independent processes. For example, we found modest but significant partial correlations of age on most validation variables after correcting for predicted disease time, with the smallest partial correlations for AD-specific biomarkers and largest partial correlations across neurodegenerative biomarkers (Supplementary Table 5). We note that correlations were calculated on a common subset of observations where all staging variables were observed to ensure comparability. Supplementary Figs 5–8 show comparisons of predicted disease time, Aβ-PET and tau-PET as staging variables for selected clinical scales and biomarkers.

**Table 2:** Spearman correlations (absolute value) between staging variables and unseen validation variables in ADNI

Domain	Validation variable	Staging variables					
		Age	Diagnosis^a^	MMSE	ADAS-cog	Aβ PET	Predicted disease time
Cognition and function	TMT-B (*n* = 2119)	0.29	0.53	0.53	**0**.**58**	0.45	0.57
	Logical memory delayed recall (*n* = 2175)	0.06	**0**.**77**	0.66	0.75	0.55	0.70
	CDR-SB (*n* = 2178)	0.11	**0**.**93**	0.68	0.74	0.65	0.82
Aβ	Plasma Aβ42/40 (*n* = 615)	0.04	0.25	0.15	0.15	**0**.**42**	0.39
	CSF Aβ42/40 (*n* = 542)	0.23	0.55	0.38	0.43	0.78	**0**.**81**
Tau	Plasma p-tau181 (*n* = 615)	0.28	0.42	0.35	0.40	0.60	**0**.**66**
	Plasma p/np-tau217 (*n* = 618)	0.14	0.56	0.45	0.50	0.78	**0**.**81**
	CSF p-tau181 (*n* = 1340)	0.15	0.48	0.39	0.44	0.58	**0**.**60**
	Tau PET Braak III-IV SUVR (*n* = 661)	0.08	0.56	0.38	0.54	0.60	**0**.**64**
Neurodegeneration	Plasma NfL (*n* = 615)	**0**.**51**	0.24	0.22	0.30	0.27	0.41
	MRI hippocampus volume (*n* = 1802)	0.38	0.57	0.51	0.58	0.43	**0**.**59**
	MRI ventricle volume (*n* = 1761)	**0**.**45**	0.29	0.29	0.35	0.26	0.38
	MRI AD thickness signature (*n* = 875)	0.34	0.55	0.55	0.62	0.43	**0**.**65**
	FDG PET SUVR (*n* = 1045)	0.13	0.57	0.55	0.65	0.44	**0**.**67**
Domain-weighted average		0.20	0.53	0.43	0.49	0.54	**0**.**63**

Correlations are computed on the subset of data with complete data for all staging variables. *n* denotes the number of observations of the validation variable. Bold text indicates the strongest correlation across staging variables. Aβ = amyloid-β; AD = Alzheimer’s disease, ADAS-cog = Alzheimer's Disease Assessment Scale-Cognitive Subscale; ADNI = Alzheimer’s Disease Neuroimaging Initiative; CDR-SB = Clinical Dementia Rating-Sum of Boxes; MCI = mild cognitive impairment; MMSE = Mini-Mental State Examination; TMT-B = Trail Making Test B; SUVR = standardized uptake value ratio.

^a^Diagnosis coded numerically as 0 = cognitively unimpaired, 1 = MCI, 2 = dementia.

**Table 3:** Spearman correlations (absolute value) between staging variables and unseen validation variables in BioFINDER

Domain	Validation variable	Staging variables					
		Age	Diagnosis^a^	MMSE	ADAS-cog	Aβ PET	Predicted disease time
Cognition and function	TMT-B (*n* = 1431)	0.45	0.49	0.45	**0**.**52**	0.45	0.49
	CDR-SB (*n* = 328)	0.02	**0**.**89**	0.63	0.75	0.67	0.79
Aβ	Plasma Aβ42/40 (*n* = 487)	0.16	0.33	0.18	0.35	0.49	**0**.**52**
	CSF Aβ42/40 (*n* = 1091)	0.25	0.53	0.33	0.46	0.78	**0**.**81**
Tau	Plasma p-tau181 (*n* = 763)	0.45	0.34	0.28	0.38	**0**.**50**	0.46
	Plasma p/np-tau 217 (*n* = 761)	0.32	0.57	0.35	0.49	**0**.**78**	0.76
	CSF p-tau181 (*n* = 1095)	0.31	0.45	0.32	0.45	0.60	**0**.**61**
	CSF p-tau 217 (*n* = 269)	0.06	0.63	0.51	0.63	0.81	**0**.**81**
	CSF p-tau 231 (*n* = 440)	0.39	0.54	0.37	0.51	0.71	**0**.**75**
	Tau PET Braak III-IV SUVR (*n* = 1250)	0.31	0.51	0.37	0.46	0.58	**0**.**58**
Neurodegeneration	Plasma NfL (*n* = 236)	0.27	0.24	0.17	0.19	0.20	**0**.**28**
	CSF NfL (*n* = 728)	0.51	0.43	0.36	0.46	0.49	**0**.**51**
	CSF neurogranin (*n* = 726)	0.18	0.26	0.24	0.30	0.35	**0**.**38**
	MRI hippocampus volume (*n* = 1226)	0.53	0.48	0.39	**0**.**55**	0.47	0.50
	MRI ventricle volume (*n* = 1226)	**0**.**57**	0.33	0.28	0.40	0.33	0.35
	MRI AD thickness signature (*n* = 1226)	0.46	0.48	0.36	0.48	0.47	**0**.**52**
Domain-weighted average		0.29	0.50	0.37	0.48	0.56	**0**.**60**

Correlations are computed on the subset of data with complete data for all staging variables. *n* denotes the number of observations of the validation variable. Bold text indicates the strongest correlation across staging variables. AD = Alzheimer's disease; ADAS-cog = Alzheimer’s Disease Assessment Scale-Cognitive Subscale; CDR-SB = Clinical Dementia Rating-Sum of Boxes; MMSE = Mini-Mental State Examination; MCI = mild cognitive impairment; TMT-B = Trail Making Test B; SUVR = standardized uptake value ratio.

^a^Diagnosis coded numerically as 0 = cognitively unimpaired, 1 = MCI, 2 = dementia.

### Biomarker trajectories

**Figure 3:**
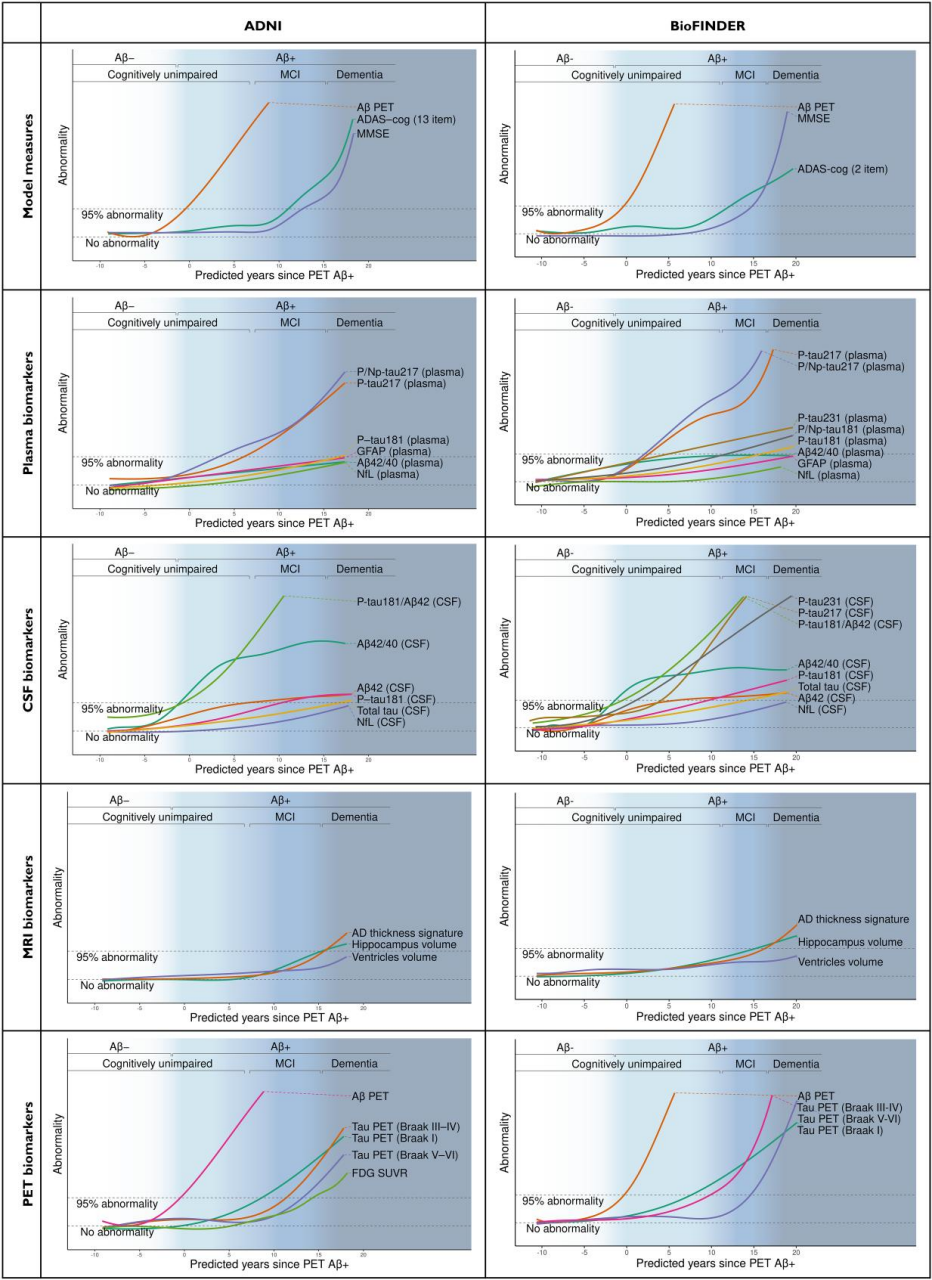
**Biomarker trajectories showing abnormality relative to cognitively unimpaired Aβ-negative subjects.** Figure shows measures included in the disease progression model, plasma biomarkers, CSF biomarkers, MRI biomarkers and PET biomarkers. Aβ = amyloid-β; ADAS-cog = Alzheimer's Disease Assessment Scale-Cognitive Subscale; MCI = mild cognitive impairment; MMSE = Mini-Mental State Examination; SUVR = standardized uptake value ratio.

**Figure 4:**
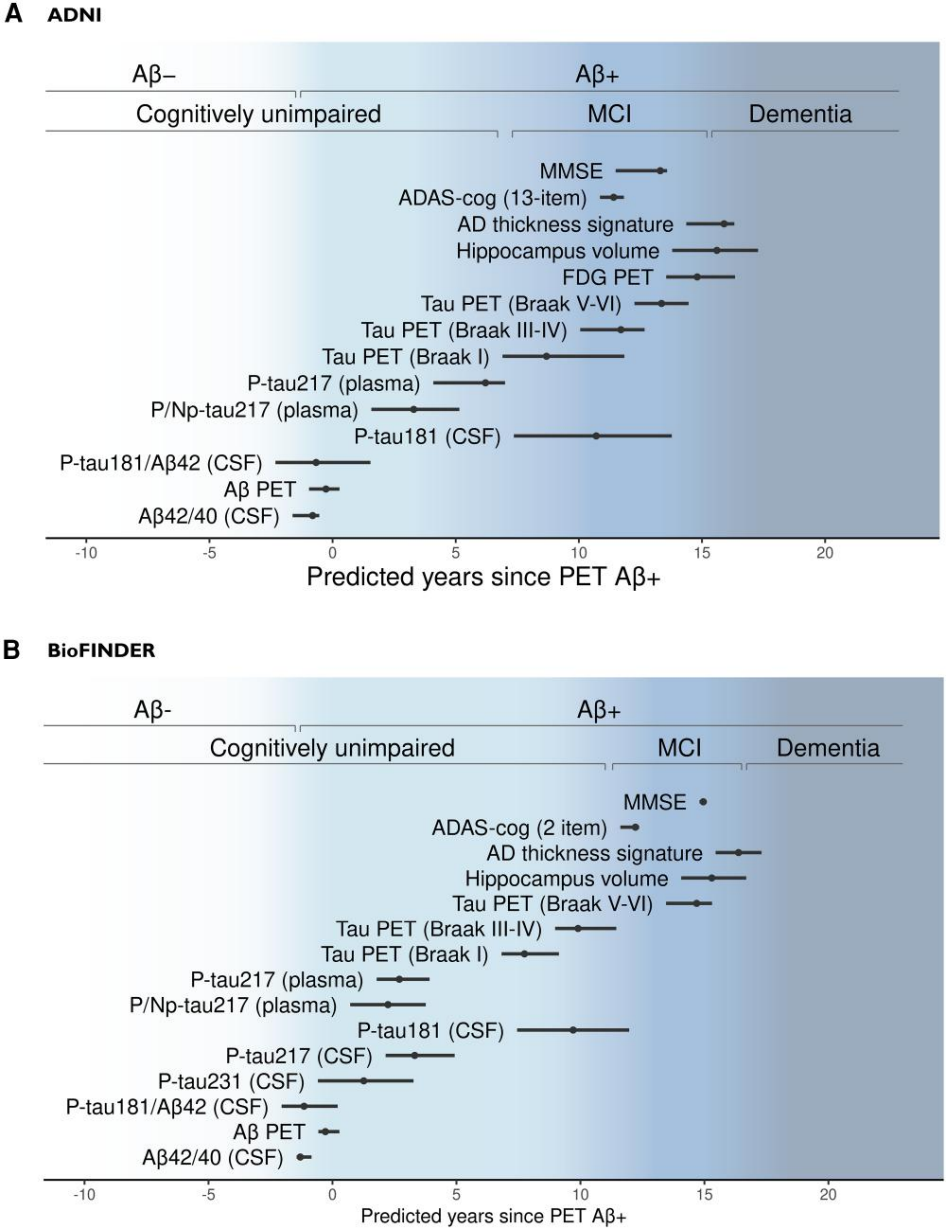
**Estimated time point at which different measures on average reach 95% abnormality threshold relative to cognitively unimpaired Aβ-negative subjects**. (**A**) ADNI and (**B**) BioFINDER. Lines represent 95% confidence intervals computed using the empirical case bootstrap. Aβ = amyloid-β; AD = Alzheimer’s disease; ADNI = Alzheimer’s Disease Neuroimaging Initiative; ADAS-cog = Alzheimer's Disease Assessment Scale-Cognitive Subscale; MCI = mild cognitive impairment; MMSE = Mini-Mental State Examination.

For the biomarkers specified in Table 4, longitudinal models for the biomarker trajectories as a function of predicted disease time were fitted. The trajectories were normalized against the median and 95% abnormality quantile for Aβ− CU, to investigate the AD-specific abnormality trajectories. Estimated biomarker abnormality trajectories generally showed consistent patterns across cohorts (Fig. 3 and Supplementary Fig. 9), and the time at which biomarkers reached abnormality relative to predicted disease time was highly consistent across both cohorts (Fig. 4). CSF Aβ_42_/Aβ_40_ reached 95% abnormality approximately 1 year prior to predicted time of Aβ-PET positivity and was largely consistent with CSF p-tau181/Aβ_42_ time of abnormality, but not CSF p-tau181 alone (abnormal 10 years after predicted predicted Aβ PET positivity). CSF p-tau231 and p-tau217, that were only available in BioFINDER, were found to reach 95% abnormality 1 and 3 years after predicted Aβ PET positivity, respectively. Plasma p/np-tau217 reached the 95% abnormality threshold 2–3 years after predicted Aβ PET positivity. In BioFINDER, plasma p-tau217 behaved very similarly to p/np-tau217, while in ADNI, plasma p-tau217 showed approximately 3 years delay in reaching 95% abnormality compared to plasma p/np-tau217. Tau-PET in Braak regions I, II-IV and V-VI reached this abnormality threshold, respectively, 7–9 years, 10–12 years and 13–15 years after Aβ-PET positivity. ADAS-cog and MMSE both became abnormal during the MCI stage of disease (11–15 years after predicted Aβ PET positivity), while volumetric MRI measures of hippocampus and cortical thickness only reached 95% abnormality in the dementia stages of disease (15–16 years after predicted Aβ PET positivity). Sensitivity analyses to assess the impact of different biomarker availability within patients on the estimated abnormality of tau biomarkers found highly consistent patterns of pairwise abnormality timings between biomarkers on subsets of patients with both tau biomarkers available and only limited numerical differences in estimates of abnormality timings based on ADNI data (Supplementary material).

**Table 4:** Available longitudinal biomarker data and correlation to predicted disease time in the ADNI and BioFINDER cohorts

	ADNI			BioFINDER		
	Measurements	Subjects	Spearman ρ with predicted disease time	Measurements	Subjects	Spearman ρ with predicted disease time
**Model measures**						
Aβ PET	2288	1138	0.82	2040	1339	0.83
ADAS-cog	6237	1447	0.82	6472	2071	0.73
MMSE	6322	1448	−0.76	6840	2088	−0.72
**Plasma**						
Aβ_42/40_	687	233	−0.38	638	638	−0.51
P-tau181	686	233	0.66	1006	1006	0.59
P/Np-tau181	–	–	–	1006	1006	0.66
P-tau217	690	233	0.80	1004	1004	0.83
P/Np-tau217	690	233	0.81	1004	1004	0.84
P-tau231	–	–	–	922	922	0.66
NfL	686	223	0.42	1303	505	0.27
GFAP	684	233	0.54	943	943	0.53
**CSF**						
Aβ_42_	2283	1213	−0.69	2702	1888	−0.71
Aβ_42/40_	684	416	−0.80	2702	1888	−0.78
P-tau181/Aβ_42_	2283	1213	0.78	2707	1893	0.63
P-tau181	2283	1213	0.57	2707	1893	0.63
P-tau217	–	–	–	1594	799	0.74
P-tau231	–	–	–	610	610	0.80
Total tau	2284	1213	0.53	2707	1893	0.58
NfL	325	325	0.42	2252	1438	0.54
Neurogranin	325	325	0.33	2252	1436	0.54
YKL-40	463	121	0.12	2254	1440	0.29
sTREM2	1275	745	−0.01	2255	1441	0.15
**MRI**						
Ventricles volume	5343	1411	0.39	2080	1258	0.44
Hippocampus volume	5106	1393	−0.63	2080	1258	−0.62
AD thickness signature	3781	839	−0.68	2080	1258	−0.66
**PET**						
Aβ PET SUVR	2288	1138	0.82	2040	1339	0.83
Tau PET SUVR (Braak I)	891	533	0.71	2089	1254	0.75
Tau PET SUVR (Braak III-IV)	891	533	0.66	2089	1254	0.72
Tau PET SUVR (Braak V-VI)	891	533	0.52	2089	1254	0.55
FDG PET SUVR	1987	976	−0.68	–	–	–

AD = Alzheimer's disease; ADAS-cog = Alzheimer’s Disease Assessment Scale-Cognitive Subscale; CDR-SB = Clinical Dementia Rating-Sum of Boxes; MMSE = Mini-Mental State Examination; np-tau = neuritic plaque tau; p-tau = phosphorylated tau; SUVR = standardized uptake value ratio.

## Discussion

In this study, we used latent-time disease progression modelling of Aβ PET and cognitive scale scores to predict years since Aβ PET positivity for subjects in ADNI and BioFINDER. The predicted disease time was shown to outperform other clinical scales, biomarkers and biomarker clocks that are often used for disease staging, including clinical diagnosis, MMSE, amyloid clock and tau clock, in terms of overall strength of correlation to unseen clinical scores and biomarkers representing Aβ, tau and neurodegeneration. Predicted disease time was also shown to produce numerically stronger correlations to the validation variables than alternative disease staging models with access to the same information, but the difference did not reach statistical significance compared to the GRACE model. These findings are consistent with those of Kühnel and colleagues,^56^ who found that a similar non-linear mixed-effects disease progression model produced significantly better predictions of future cognitive trajectories than LTJMM and GRACE.

Compared to conventional staging approaches, predicted disease time has the advantage that a prediction based on any set of observed cross-sectional or longitudinal data will enable calculation of predicted disease time at any future visit, while some staging measures, such as clinical scales or PET-based biomarkers, may be difficult to project to visits where they were not assessed. Amyloid and tau PET clocks offer an alternative solution to this problem, but as demonstrated here, these biomarker clocks produce less generalizable stagings than our proposed model. The difference which may be caused by the clocks capturing a single aspect of the disease, that there may be ranges of Aβ and tau PET quantifications that have low predictive value for disease staging (e.g. values below abnormality thresholds, Aβ PET in later symptomatic disease stages) and that the clocks are more affected by noise in the biomarker data due to the more direct translations. Recent work has demonstrated the feasibility of estimating typical biomarker profiles associated with continuous-time disease stage from latent-time disease progression modelling, which in turn enable improved prognostication based on a collection of biomarkers measured at a single visit that reflect different aspects of AD.^4^

Based on predicted years since Aβ PET positivity, biomarker trajectories were estimated on a joint timescale, and the abnormality of individual biomarkers along the disease timeline were analysed. This provided new insights by estimating the temporal relationships between when biomarkers and clinical outcome measures typically become abnormal, and the temporal relationships between markers of insoluble and soluble pathology. The trajectories of imaging biomarkers were well aligned with the amyloid cascade hypothesis, suggesting that the typical evolution of biomarker profiles along the AD trajectory is one where Aβ biomarkers initially become abnormal, followed by abnormal tau biomarkers and finally abnormal neurodegeneration biomarkers. However, it was found that that amyloidopathy defined using a biofluid-based biomarker (CSF Aβ_42_/Aβ_40_) was detectable prior to Aβ PET abnormality, which is in agreement with previous results comparing CSF and PET Aβ biomarkers.^36^ Further, we found that some biomarkers of soluble phosphorylated tau became detectably abnormal 1–3 years after Aβ biomarkers (CSF p-tau231, CSP p-tau217, plasma p/np-tau217), many years earlier than tau PET signals became abnormal. P-tau biomarkers (especially p-tau231 but to some extent also p-tau217) have been shown to be very closely associated with early Aβ accumulation,^42,60,61^ so the observed signal could reflect early Aβ-induced changes in p-tau and not insoluble tau pathology. Compared to CSF p-tau231 and p-tau217, p-tau181 increased considerably later, reaching 95% abnormality approximately 10 years after Aβ PET. This difference may be partly explained by the effect of different assays analytical protocols for p-tau181 compared to p-tau231 and p-tau217.^8,62^ The findings of the present study are largely in agreement with recent findings by Jia and colleagues^63^ in a Chinese cohort with 20 years follow-up. In particular, the Chinese study suggested that CSF Aβ-biomarkers became abnormal 14–18 years before diagnosis and CDR-SB scores becoming abnormal 6 years prior to diagnosis, which is consistent with the 12-year gap between abnormality of CSF Aβ_42_/Aβ_40_ and ADAS-cog in the current study. Differences in timing of abnormality of other CSF markers such as p-tau181 relative to CSF amyloid positivity (3–7 years in Chinese study, 11 years in the present study) may reflect differences in assays, definition of abnormality thresholds and other methodological differences (including use of imputation of biomarker values in the study by Jia *et al*.^63^).

An important consideration for the estimated time course of biomarker changes presented here is that it does not directly reflect the biological progression of disease but is also influenced by the sensitivity and stability of the biomarker and the natural variation of the biomarker in non-AD populations. The latter feature means that biomarkers that are not specific to AD, such as the neurodegenerative biomarkers considered here, will appear less anomalous than biomarkers more closely related to AD pathology, but may still track disease progression well within a population of AD patients.

Overall, we showed that despite differences in assays, tracers and processing methods, AD progression was associated with a highly consistent pattern of biomarker progression across two separate cohorts. With the availability of the first approved disease-modifying therapies for AD in the form of high-clearance Aβ-targeting immunotherapies, biomarkers will play an increasingly important role in verification of the presence of Aβ pathology and early identification of patients. Biomarker-based staging of patients has already been implemented in some clinical trials through tau-PET-based inclusion criteria,^64,65^ in an effort to exclude patients who are early on the disease continuum and thus unlikely to decline during the study period (thus masking a treatment effect) or patients who are late on the disease continuum and thus may be too advanced to fully benefit of the treatment. A natural hypothesis being tested in several large studies is that Aβ-targeting immunotherapies would be most efficacious if delivered in the earliest stages of AD, when little tau pathology is present.^66^ Resultingly, it is important to know the typical duration of elevated Aβ plaque load without elevated tau. Recently, Therneau and colleagues^67^ used an accelerated failure time model with similarities to the approach presented here to estimate the temporal relationship between Aβ-PET and tau-PET. They found the average delay between when Aβ-PET and tau-PET would reach a change point and begin to increase abnormally to be 13.3 years. This duration is slightly longer than the difference between when Aβ-PET and tau-PET became abnormal in the present analysis, with differences of 9 years (Braak I) and 12 years (Braak III-IV) in ADNI and 8 years (Braak I) and 10 years (Braak III-IV) in BioFINDER. The difference in the type of events studied (change in accumulation versus abnormality relative to Aβ− CU) may have contributed to the differences.

Our work has some limitations. Staging of patients was achieved by modelling under certain assumptions. A key assumption was that AD can be described as evolving around a single multivariate trajectory on a single timescale. However, there may exist AD subtypes with distinct trajectories,^68^ and rate of decline and cognitive manifestation can be affected by patient characteristics such as age, comorbidities and co-pathologies.^4,26,32^ In particular, we found that age had substantial partial correlations with neurodegeneration biomarkers after controlling for predicted disease time (Supplementary Table 5). In the present study, such variation was captured by random effects or measurement noise terms. More elaborate modeling of differences in rate of decline and systematic deviations could yield more precise estimates of biomarker evolution. Another assumption of the model was that missing data was not informative, but since patients are more likely to drop out of the study as disease progresses, the disease progression model may rely on a healthier group of subjects and thus estimate a longer disease duration in the later stages of disease than that typically seen in the real world.^69^

In conclusion, this study used latent-time modellling to analyse longitudinal data from two large cohorts of subjects that were well characterized in terms of their AD status. The continuous-time staging of patients based on the disease progression model was shown to be superior to existing methods and by analysing biomarker trajectories along the resulting AD continuum, we believe our study offers the most accurate estimates of the temporal progression of AD pathology to date.

## Supplementary Material

awaf413_Supplementary_Data

## Data Availability

ADNI data is available to qualified academic investigators submitting an online application for access. For more information, please see the ADNI website http://adni.loni.usc.edu/. Pseudonymized data from BioFINDER will be made available by request from a qualified academic investigator for the sole purpose of replicating procedures and results presented in the article and if data transfer is in agreement with EU legislation on the general data protection regulation and decisions by the Ethical Review Board of Sweden and Region Skåne, which should be regulated in a material transfer agreement.
