# Supplementary material for: Estimating the time course of biomarker changes in Alzheimer’s disease
Source: Brain. 2025 Nov 3;149(6):1929–43. doi: 10.1093/brain/awaf413 (PMC13232038; doi:10.1093/brain/awaf413)
Supplement: awaf413_Supplementary_Data [file awaf413_supplementary_data.pdf]

Supplementary Material

Estimating the time course of biomarker changes in Alzheimer's disease

Lars Lau Raket[1,2], Alexa Pichet Binette[1], Niklas Mattsson-Carlgren[1,3,4], Shorena Janelidze[1], for the Alzheimer's Disease Neuroimaging Initiative,* Henrik Zetterberg[5,6,7,8,9,10], Nicholas J. Ashton[5,11,12], Kaj Blennow[5,6], Erik Stomrud[1,13], Sebastian Palmqvist[1,13] and Oskar Hansson[1,13]

1. Diagnostic proportions along disease continuum

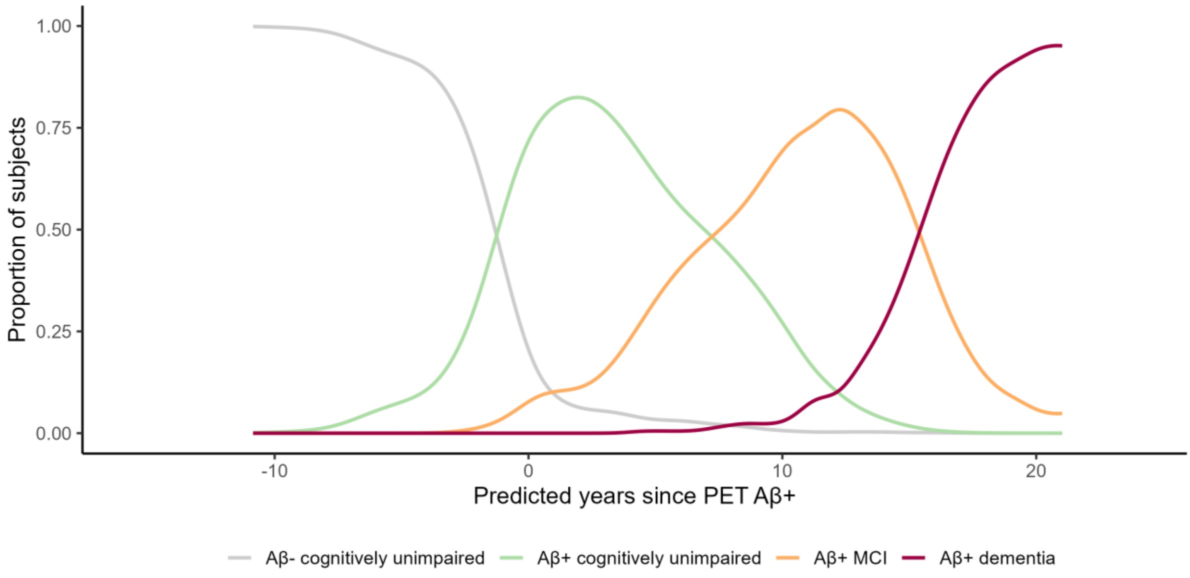

Figure S1 ADNI diagnostic proportion along predicted disease continuum.

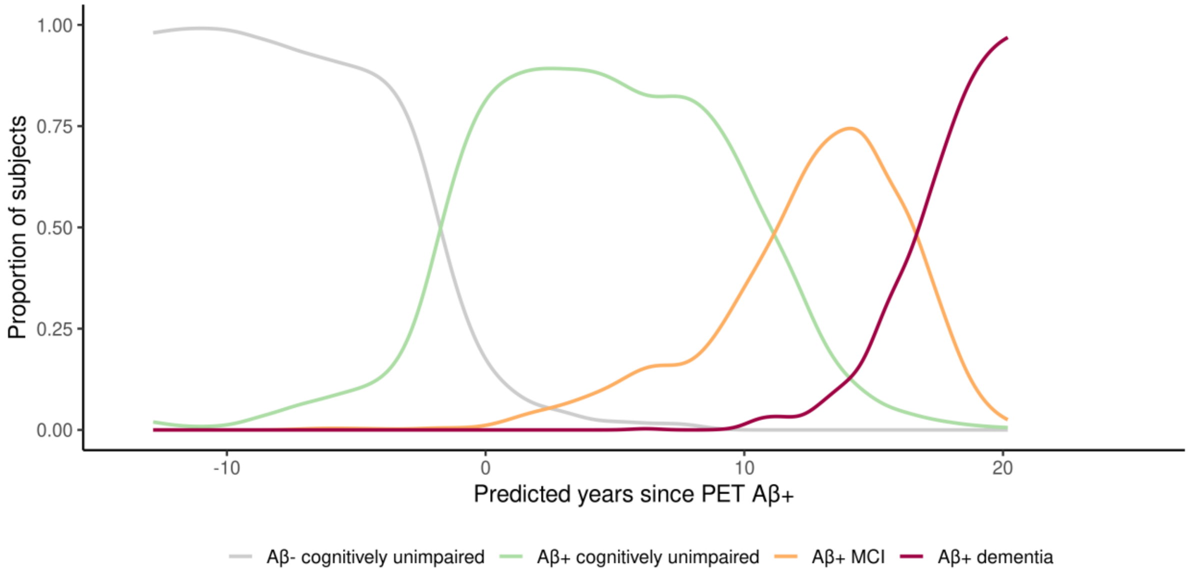

Figure S2 BioFINDER diagnostic proportion along predicted disease continuum.

2. Model diagnostics

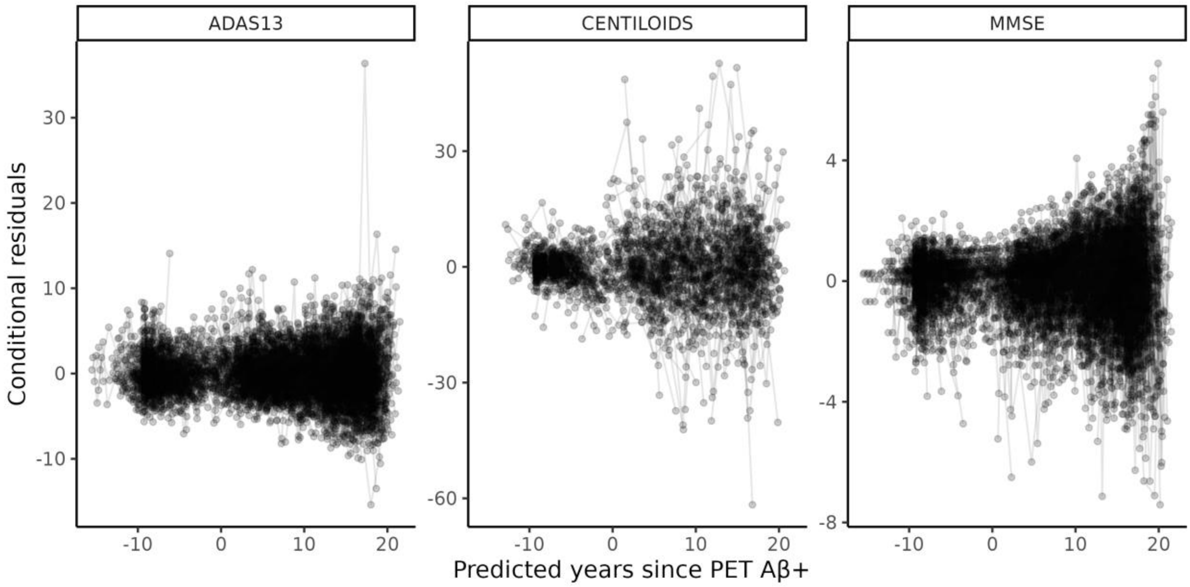

Figure S3 Conditional residuals in ADNI plotted against predicted years since PET Aβ+.

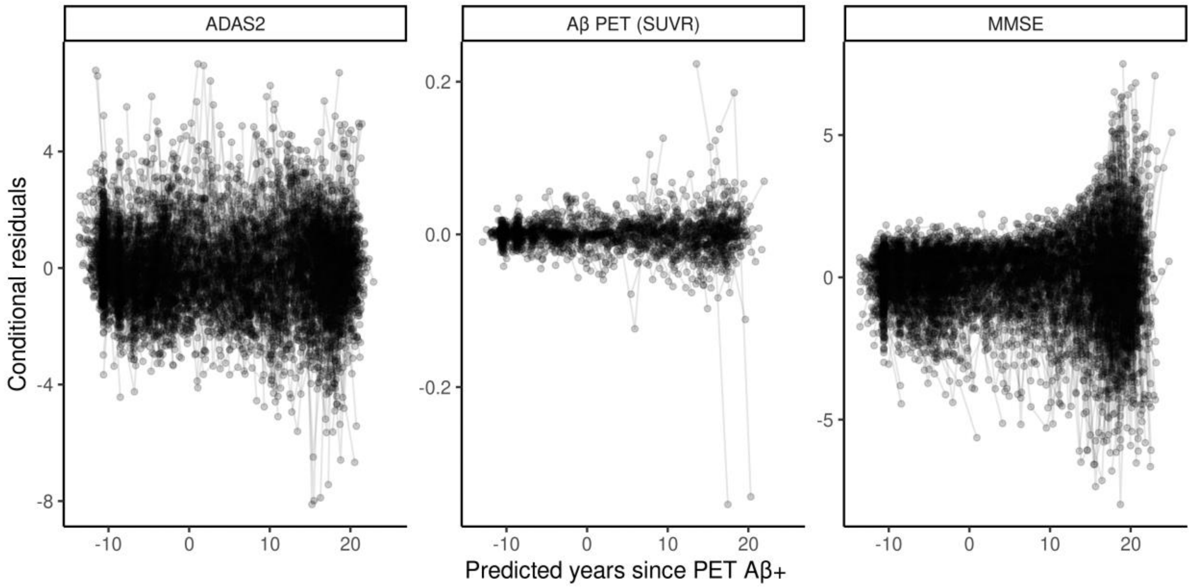

Figure S4 Conditional residuals in BioFINDER plotted against predicted years since PET Aβ+.

3. Variance explained

Table S1 Comparison of properties of the full model with sub-models excluding random effects when fitted on the ADNI dataset

			Estimated standard deviations for random variables			
**Model**	**Degrees of freedom**	**Log likelihood**	s_i	x_i	e_{ijk}	$x_i + e_{ijk}$
**Full model**	30	-41540.97	3.24	Centiloid 24.8<br>ADAS-cog 4.43<br>MMSE 1.20	Centiloid 12.8<br>ADAS-cog 3.34<br>MMSE 1.49	Centiloid 27.9<br>ADAS-cog 5.55<br>MMSE 1.91
**Exclude x_i**	24	-43213.00	3.35	—<br>—<br>—	Centiloid 27.5<br>ADAS-cog 4.54<br>MMSE 1.71	Centiloid 27.5<br>ADAS-cog 4.54<br>MMSE 1.71
**Exclude s_i**	29	-44871.57	—	Centiloid 28.6<br>ADAS-cog 6.46<br>MMSE 2.01	Centiloid 13.4<br>ADAS-cog 4.87<br>MMSE 2.16	Centiloid 31.6<br>ADAS-cog 8.09<br>MMSE 2.95
**Exclude x_i and s_i**	23	-49253.18	—	—<br>—<br>—	Centiloid 20.5<br>ADAS-cog 7.47<br>MMSE 3.31	Centiloid 20.5<br>ADAS-cog 7.47<br>MMSE 3.31

Table S2 Comparison of properties of the full model with sub-models excluding random effects when fitted on the BioFINDER dataset

			Estimated standard deviations for random variables			
**Model**	**Degrees of freedom**	**Log likelihood**	s_i	x_i	e_{ijk}	$x_i + e_{ijk}$
**Full model**	29	-27700.61	3.85	Aβ SUVR 0.158<br>ADAS-cog 2.16<br>MMSE 0.647	Aβ SUVR 0.040<br>ADAS-cog 1.77<br>MMSE 1.58	Aβ SUVR 0.163<br>ADAS-cog 2.80<br>MMSE 1.71
**Exclude x_i**	23	-29565.78	4.49	—<br>—<br>—	Aβ SUVR 0.162<br>ADAS-cog 2.39<br>MMSE 1.67	Aβ SUVR 0.162<br>ADAS-cog 2.39<br>MMSE 1.67
**Exclude s_i**	28	-29877.68	—	Aβ SUVR 0.195<br>ADAS-cog 2.65<br>MMSE 2.10	Aβ SUVR 0.042<br>ADAS-cog 1.87<br>MMSE 2.21	Aβ SUVR 0.199<br>ADAS-cog 3.24<br>MMSE 3.05
**Exclude x_i and s_i**	22	—	—	—<br>—<br>—	Aβ SUVR —<br>ADAS-cog —<br>MMSE —	Aβ SUVR —<br>ADAS-cog —<br>MMSE —

Estimates from the model without random effects are not reported since model fitting did not converge

4. Correlation analysis of predicted disease time and alternative staging methods on unseen validation variables in ADNI

Table S3 Spearman correlations (absolute value) between predicted disease time, amyloid PET clock, and tau PET clock, and unseen validation variables in ADNI.

Domain	Validation variable	n	Amyloid clock	Predicted disease time	n	Tau clock	Predicted disease time
Cognition and function	Trail making B	4447	0.44	**0.57**	1937	0.30	**0.51**
	Logical memory delayed recall	3952	0.52	**0.71**	1809	0.46	**0.56**
	CDR-SB	4769	0.64	**0.84**	2045	0.54	**0.72**
Aβ	Plasma Aβ42/40	609	0.77	**0.80**	502	0.52	**0.80**
	CSF Aβ42/40	655	0.39	0.38	393	0.19	**0.37**
Tau	Plasma p-tau181	654	0.60	**0.64**	392	0.40	**0.59**
	Plasma p/np-tau217	658	0.78	**0.81**	394	0.51	**0.75**
	CSF p-tau181	1589	0.55	**0.59**	711	0.45	**0.54**
	Tau PET Braak III-IV SUVR	828	0.57	**0.63**	—	—	—
Neurodegeneration	Plasma NfL	654	0.34	**0.42**	392	0.23	**0.38**
	MRI hippocampus volume	3877	0.42	**0.59**	1561	0.36	**0.48**
	MRI ventricle volume	3894	0.27	**0.36**	1576	0.22	**0.36**
	MRI AD thickness signature	2559	0.40	**0.59**	580	0.10	**0.21**
	FDG PET SUVR	1280	0.44	**0.65**	374	0.33	**0.54**
**Domain-weighted average**			0.53	**0.62**		0.37	**0.55**

Correlations are computed on the subset of data with complete data for both the biomarker clock and predicted disease time

n denotes number of observations of the validation variable

Bold text indicates the strongest correlation across staging variables

Table S4 Spearman correlations (absolute value) between predicted disease time, disease time estimated by GRACE and LTJMM, and unseen validation variables in ADNI.

		Staging method		
**Domain**	**Validation variable**	**GRACE disease time**	**LTJMM disease time**	**Predicted disease time**
**Cognition and function**	Trail making B (*n* = 6034)	**0.68**	0.58	0.63
	Logical memory delayed recall (*n* = 5220)	**0.81**	0.65	0.76
	CDR-SB (n = 6491)	0.84	0.73	**0.86**
**Aβ**	Plasma Aβ42/40 (*n* = 687)	0.29	0.13	**0.38**
	CSF Aβ42/40 (*n* = 684)	0.63	0.51	**0.80**
**Tau**	Plasma p-tau181 (*n* = 686)	0.57	0.46	**0.66**
	Plasma p/np-tau217 (n = 690)	0.70	0.45	**0.81**
	CSF p-tau181 (*n* = 2283)	0.53	0.48	**0.57**
	Tau PET Braak III-IV SUVR (*n* = 891)	0.60	0.51	**0.66**
**Neurodegeneration**	Plasma NfL (*n* = 686)	0.36	0.39	**0.42**
	MRI hippocampus volume (*n* = 5106)	**0.65**	0.60	0.63
	MRI ventricle volume (*n* = 5343)	0.37	0.38	**0.39**
	MRI AD thickness signature (*n* = 3781)	**0.69**	0.65	0.67
	FDG PET SUVR (*n* = 1987)	0.66	0.61	**0.68**
	**Domain-weighted average**	0.59	0.49	**0.64**

Correlations are computed on the subset of data with complete data for all staging variables

n denotes number of observations of the validation variable

Bold text indicates the strongest correlation across staging variables

5. Partial correlation analysis of predicted disease time and age on unseen validation variables in ADNI

Table S5 Partial Spearman correlations between age, predicted disease time and unseen validation variables in ADNI.

Domain	Validation variable	Age	Predicted disease time
Cognition and function	Trail making B (*n* = 2119)	0.19 (p < 0.001)	0.53 (p < 0.001)
	Logical memory delayed recall (*n* = 2175)	0.15 (p < 0.001)	-0.71 (p < 0.001)
	CDR-SB (n = 2178)	-0.15 (p < 0.001)	0.82 (p < 0.001)
Aβ	Plasma Aβ42/40 (*n* = 615)	0.04 (p = 0.3525)	-0.38 (p < 0.001)
	CSF Aβ42/40 (*n* = 542)	0.08 (p = 0.0776)	-0.79 (p < 0.001)
Tau	Plasma p-tau181 (*n* = 615)	0.20 (p < 0.001)	0.64 (p < 0.001)
	Plasma p/np-tau217 (n = 618)	-0.05 (p = 0.2055)	0.81 (p < 0.001)
	CSF p-tau181 (*n* = 1340)	-0.03 (p = 0.3229)	0.59 (p < 0.001)
	Tau PET Braak III-IV SUVR (*n* = 661)	-0.20 (p < 0.001)	0.65 (p < 0.001)
Neurodegeneration	Plasma NfL (*n* = 615)	0.47 (p < 0.001)	0.36 (p < 0.001)
	MRI hippocampus volume (*n* = 1802)	-0.32 (p < 0.001)	-0.57 (p < 0.001)
	MRI ventricle volume (*n* = 1761)	0.40 (p < 0.001)	0.32 (p < 0.001)
	MRI AD thickness signature (*n* = 875)	-0.37 (p < 0.001)	-0.65 (p < 0.001)
	FDG PET SUVR (*n* = 1045)	-0.07 (p = 0.0258)	-0.67 (p < 0.001)

Correlations are computed on the subset of data with complete data for all staging variables shown in Table 2.

n denotes number of observations of the validation variable

6. Head-to-head comparisons of predicted disease time, Aβ PET, and tau

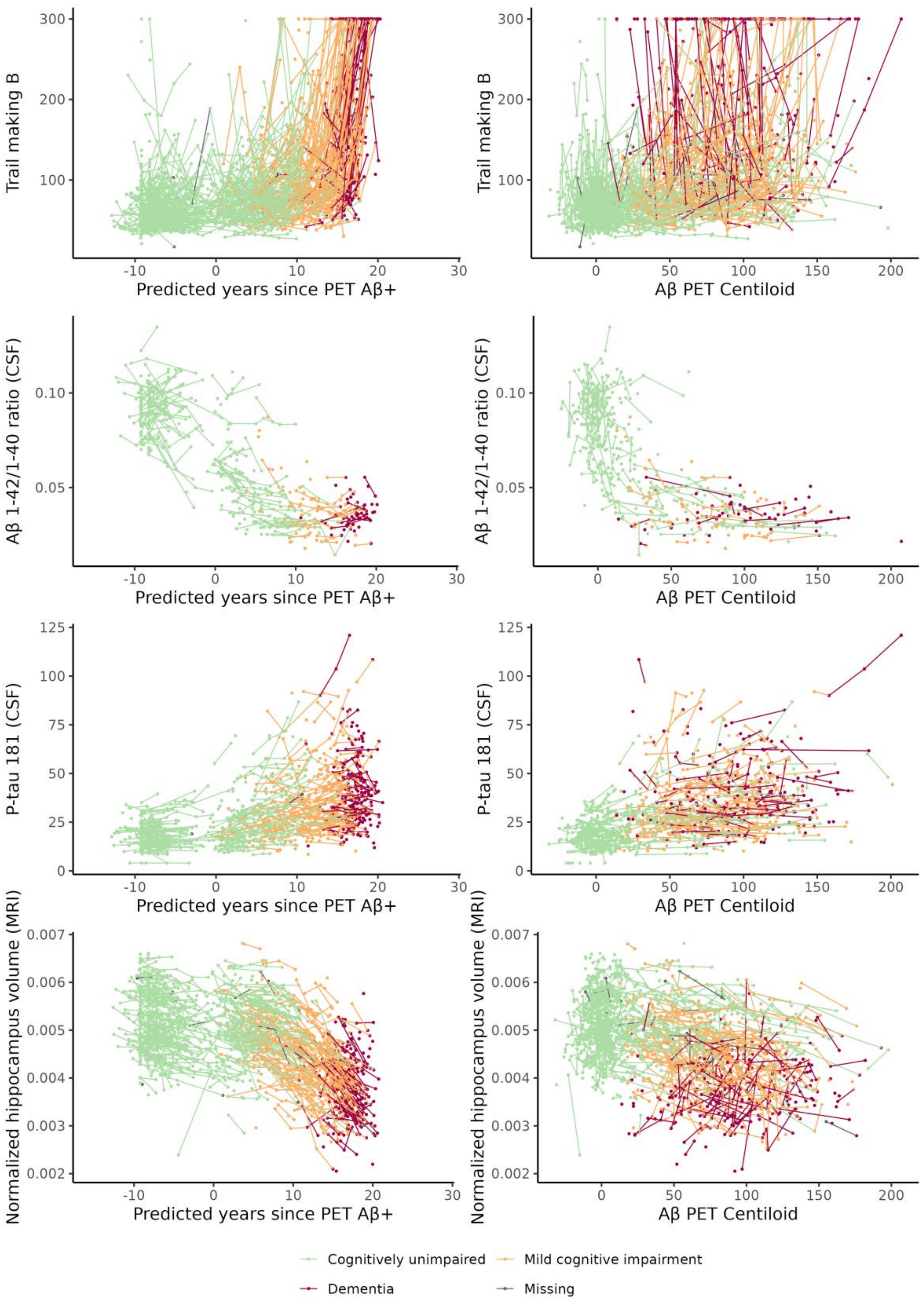

Figure S5 ADNI: Head-to-head comparison of predicted disease time and Aβ PET.

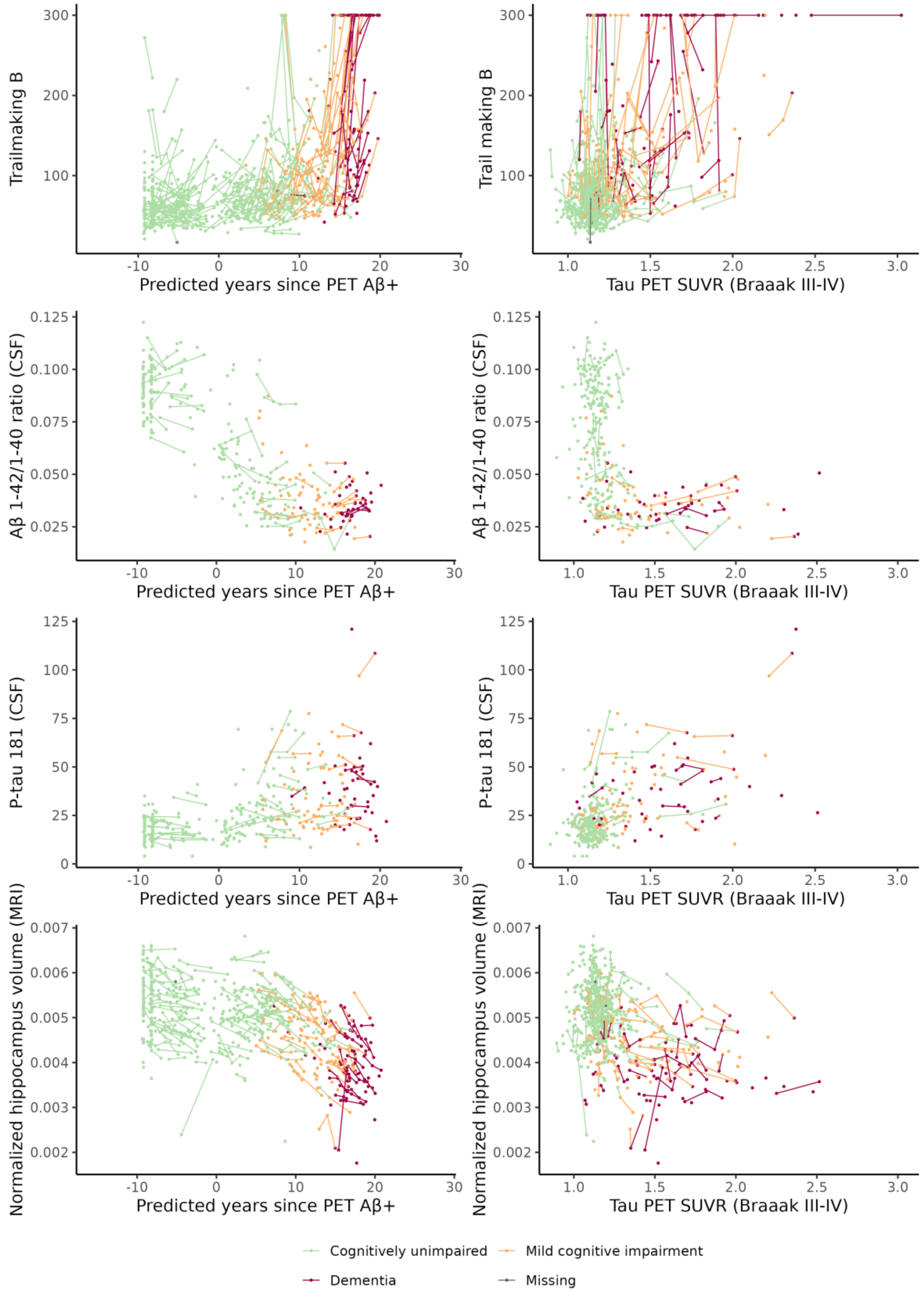

Figure S6 ADNI: Head-to-head comparison of predicted disease time and tau PET.

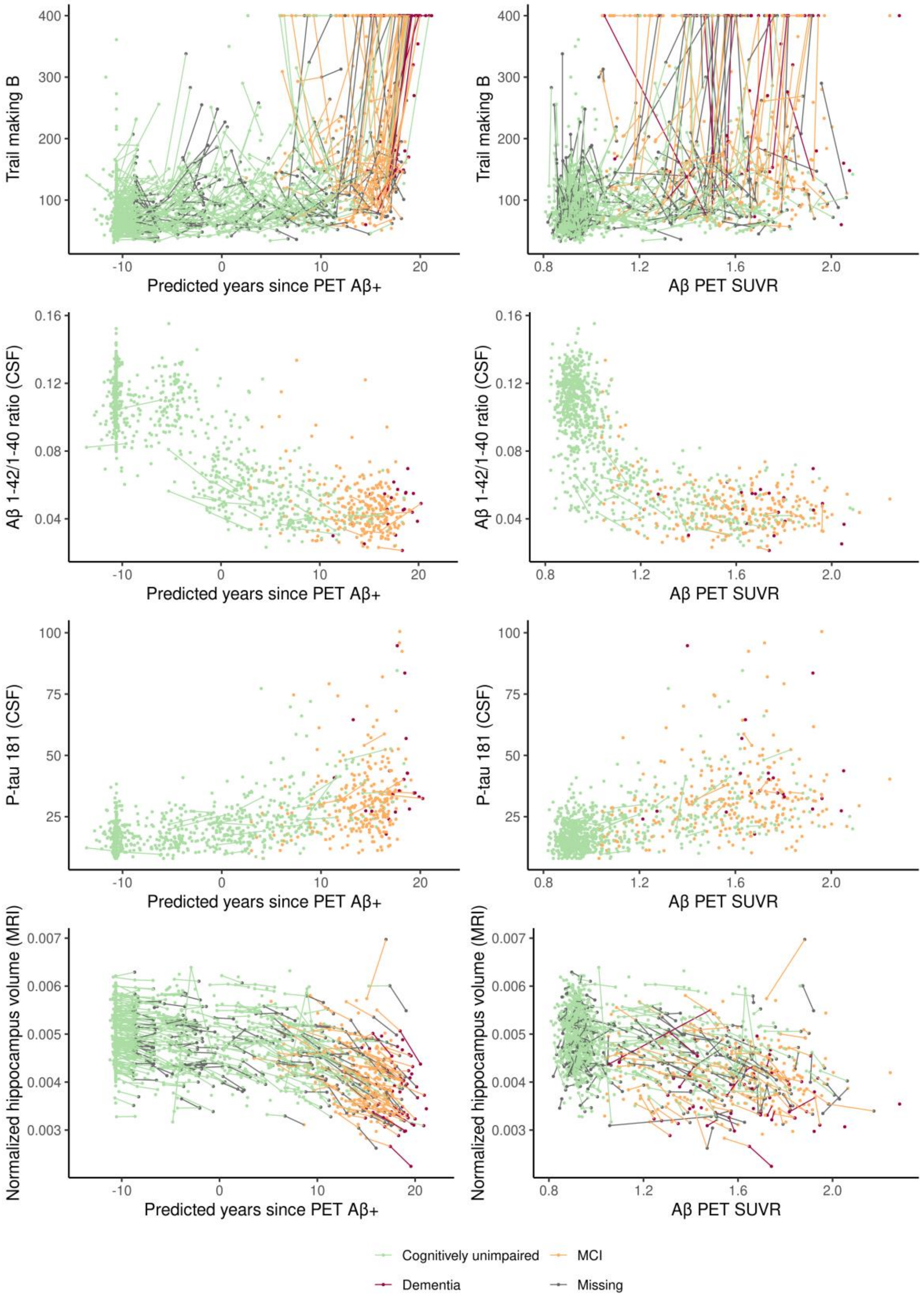

Figure S7 BioFINDER: Head-to-head comparison of predicted disease time and Aβ PET.

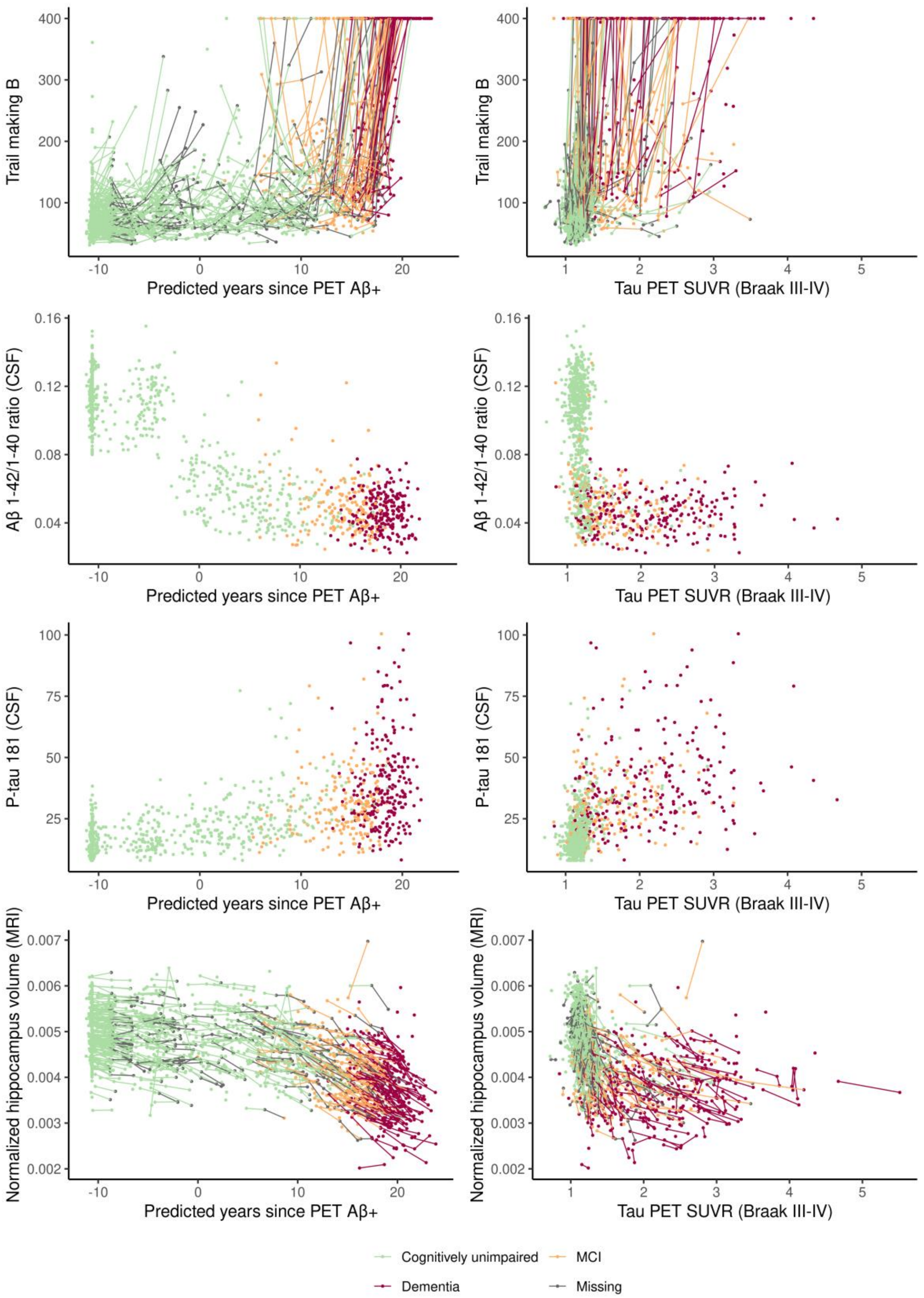

Figure S8 BioFINDER: Head-to-head comparison of predicted disease time and tau PET.

7. Abnormality trajectories of additional CSF biomarkers

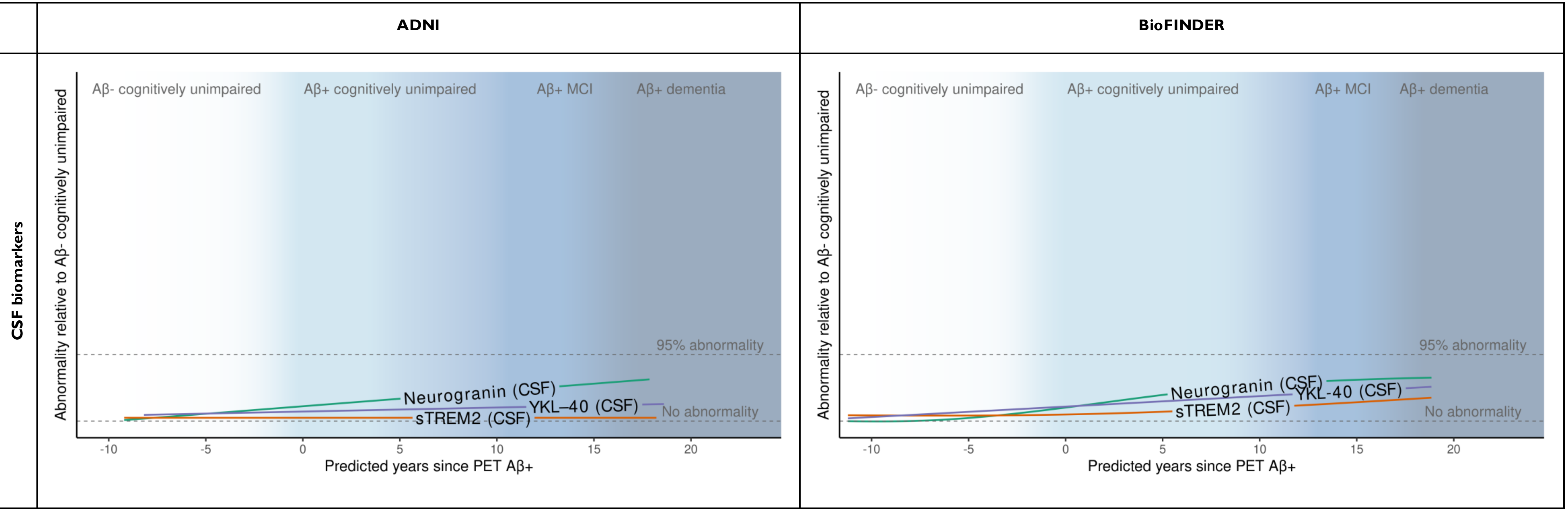

Figure S9. Additional CSF biomarker trajectories of abnormality relative to cognitively unimpaired Aβ-negative subjects for measures included in the disease progression model.

8. Comparing timing of tau biomarker abnormality in ADNI

Different types of biomarkers may not consistently be available within the same patient, and this may to some degree be confounded by the protocol under which the patient were recruited and the type of patient. To assess the impact of this on the estimated timing of biomarkers crossing the abnormality threshold in ADNI (Figure 4A), we conducted sensitivity analyses, comparing the timing of abnormality of different tau biomarkers in four sets of patients.

1. **Full cohort**. Included all observations of the biomarker of interest.
2. **CSF tau subset**. Included only subjects with at least one valid assessment of P-tau181/Aβ42 in CSF.
3. **Plasma tau subset**. Included only subjects with at least one valid assessment of p/np-tau217 in plasma.
4. **Tau PET subset**. Included only subjects with at least one tau PET scan with valid SUVR quantification in Braak regions.

Results are shown in Figure S1, and while there are some numerical differences in the estimated timing of abnormality, all estimates from the full cohort fall within the 95% confidence intervals of the estimates in the three subsets.

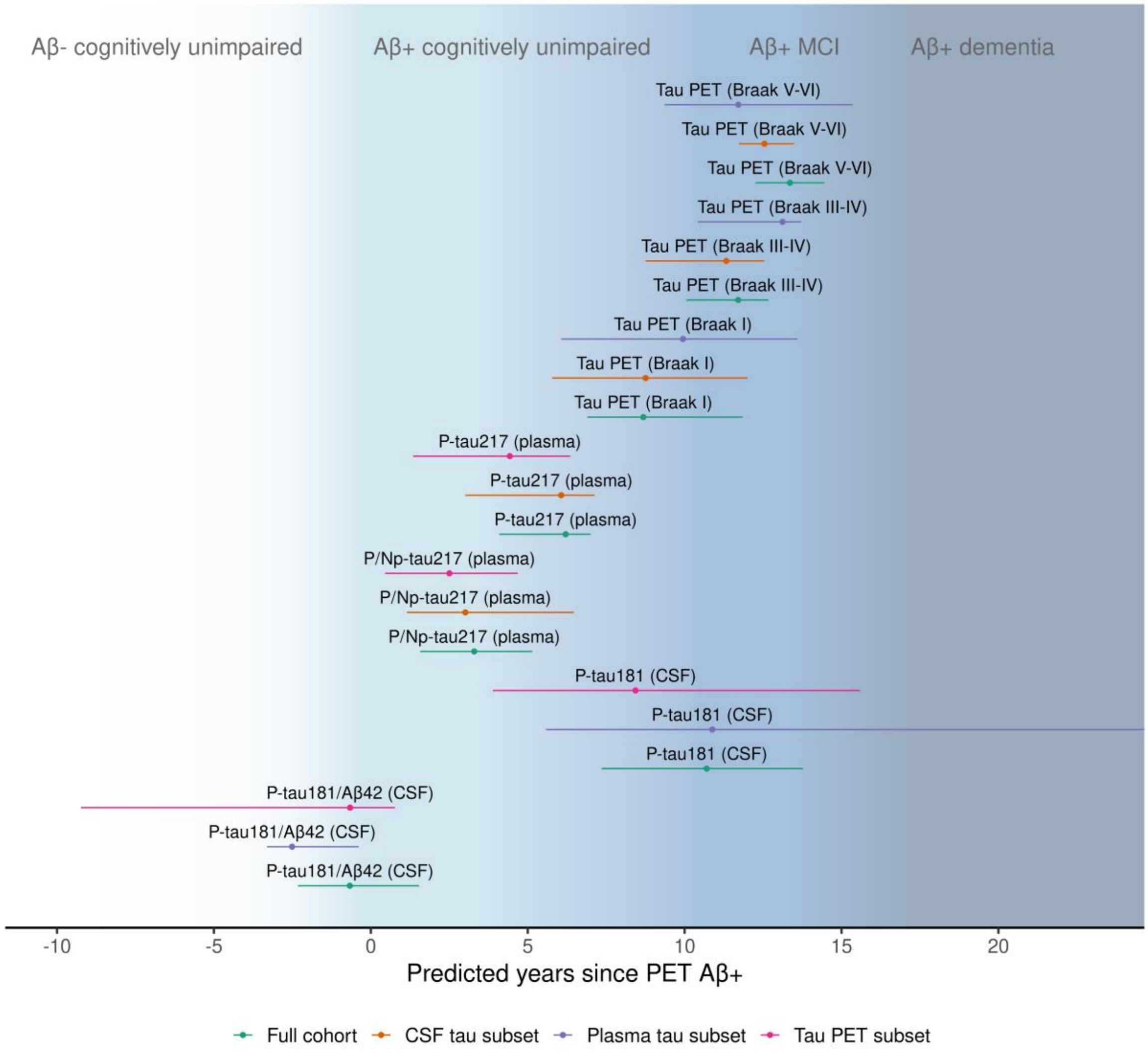

Figure S10 Estimated time point of when different tau-related biomarkers on average reach 95% abnormality threshold relative to cognitively unimpaired Aβ-negative subjects in various subsets of ADNI.
